# Genome-Wide and Locus-Level Analyses Reveal Modest, Heterogeneous Genetic Sharing Between Alzheimer’s Disease and Myasthenia Gravis

**DOI:** 10.3390/ijms27114792

**Published:** 2026-05-26

**Authors:** Emmanuel O. Adewuyi, Asa Auta, Chinedu I. Ossai, Chidozie C. Anyaegbu, Thi Thu Huong Nguyen, Md Rezanur Rahman, Blossom C. M. Stephan, Gizachew A. Tessema, Dale R. Nyholt, Gavin Pereira

**Affiliations:** 1 Curtin School of Population Health, Faculty of Health Sciences, Curtin University, Perth, WA 6102, Australia; thithuhuong.nguyen1@curtin.edu.au (T.T.H.N.); gizachew.tessema@curtin.edu.au (G.A.T.); gavin.f.pereira@curtin.edu.au (G.P.); 2 Curtin Medical Research Institute, Faculty of Health Sciences, Curtin University, Perth, WA 6102, Australia; chidozie.anyaegbu@curtin.edu.au; 3 Anchor University Centre for Global Health (AUCGH), Anchor University, Lagos 100278, Nigeria; 4 Dementia Centre of Excellence, Curtin enAble Institute, Faculty of Health, Curtin University, Perth, WA 6102, Australia; blossom.stephan@curtin.edu.au; 5 Faculty of Health, Social Care and Medicine, Edge Hill University, Ormskirk L39 4QP, UK; asa.auta@edgehill.ac.uk; 6 Independent Researcher, Melbourne, VIC 3107, Australia; ossaic@gmail.com; 7 Perron Institute for Neurological and Translational Science, Ralph and Patricia Sarich Neuroscience Research Institute, Nedlands, WA 6009, Australia; 8 Brain and Mental Health Program, QIMR Berghofer Medical Research Institute, Herston, Brisbane, QLD 4006, Australia; rezanur.rahman@qimrb.edu.au; 9 Curtin enAble Institute, Curtin University, Perth, WA 6102, Australia; 10 Centre for Genomics and Personalised Health, School of Biomedical Sciences, Faculty of Health, Queensland University of Technology, Brisbane, QLD 4000, Australia; d.nyholt@qut.edu.au; 11 Faculty of Medicine, Universitas Negeri Malang, Malang 65145, Indonesia

**Keywords:** Alzheimer’s disease, genetic overlap, immune dysregulation, locus-specific heterogeneity, myasthenia gravis

## Abstract

Alzheimer’s disease (AD) is a neurodegenerative disorder, whereas myasthenia gravis (MG) is an autoimmune neuromuscular disease. Despite their distinct clinical manifestations, both disorders involve immune dysregulation and cholinergic dysfunction, and epidemiological evidence for an association remains inconclusive. Here, we investigated the genetic architecture underlying the AD–MG relationship using large-scale European-ancestry genome-wide association study (GWAS) data, including early- and late-onset MG, within a multi-resolution analytical framework. Genome-wide analyses indicated modest polygenic overlap between AD and MG, supported by nominally significant and directionally consistent correlations across datasets, SNPeffect concordance in the primary GWAS, and robust gene-level overlap. Evidence for genome-wide correlation was weaker and non-significant across AD-MG subtypes. Local genetic correlation analyses revealed that shared AD-MG signals were largely locus-specific and heterogeneous, with regions showing both concordant and discordant effects, particularly across MG subtypes. Subtype-specific analyses indicated broader and more heterogeneous overlap for AD–late-onset MG, including both major histocompatibility complex (MHC) and non-MHC loci, whereas AD–early-onset MG showed more restricted patterns largely confined to the MHC. Cross-trait meta-analysis and colocalisation further refined these findings, identifying a limited number of loci with evidence of shared AD-MG association, while most regions were consistent with distinct causal variants. A chromosome 16 locus showed the most consistent shared cross-trait AD-MG signal across multiple analytical frameworks. Mendelian randomisation analyses provided no evidence of a causal effect of AD liability on MG and yielded only suggestive, and inconclusive evidence for the reverse direction. Gene-level and expression-informed analyses prioritised immune-related genes, as well as regulators of transcription, chromatin organisation, and synaptic processes, without implying concordant causal variants across traits. Tissue and pathway analyses suggested shared immune involvement, with differential emphasis on innate immune processes in AD and adaptive immune pathways in MG. Notably, heterogeneity of effects within the MHC and across loci suggests that overlap reflects a complex, context-dependent architecture rather than a uniform immune-driven signal. Overall, our findings indicate that the AD–MG relationship is characterised by modest genome-wide polygenic overlap, substantial locus-specific heterogeneity, and partial convergence on immune-related genetic architecture, rather than a uniformly shared mechanism.

## 1. Introduction

Alzheimer’s disease (AD) and myasthenia gravis (MG) are clinically distinct disorders. However, emerging evidence suggests potential biological overlap, with some studies also hinting at epidemiological associations [1,2,3]. AD, the most common form of dementia, is characterised by progressive cognitive decline and hallmark pathological features, including amyloid beta (Aβ) plaques and tau neurofibrillary tangles [4,5,6]. A well-established aspect of AD is the degeneration of basal forebrain cholinergic neurons, leading to reduced cortical acetylcholine levels, a key neurotransmitter involved in learning and memory [7]. The ‘cholinergic hypothesis’ suggests that this neurotransmitter deficit underlies much of the cognitive impairment observed in AD [7]. Moreover, immune dysfunction and neuroinflammation are recognised as central to AD pathogenesis [8]. In contrast, MG is a chronic autoimmune neuromuscular disorder defined by autoantibodies targeting components of the neuromuscular junction, most notably the nicotinic acetylcholine receptor (AChR) [9,10]. These autoantibodies disrupt neuromuscular transmission, leading to fluctuating skeletal muscle weakness [10,11]. MG is frequently associated with thymic abnormalities, with some studies implicating thymic Epstein–Barr virus (EBV) reactivation as a trigger for B- and T-cell-mediated autoimmunity [12,13,14], reflecting underlying immune dysregulation [11].

Despite their differing primary sites of pathology, with AD as a neurodegenerative brain disorder and MG as a systemic autoimmune condition, both involve disruptions in cholinergic signalling and immune system dysregulation. In AD, cholinergic dysfunction is associated with the loss of basal forebrain neurons, while in MG, acetylcholine signalling is impaired at the neuromuscular junction [7,11]. Notably, nicotinic AChRs are also expressed in the central nervous system [9], raising the possibility that MG-related autoimmunity could affect central cholinergic tone. This direct autoimmune targeting of cholinergic receptors distinguishes MG from most other autoimmune diseases, in which cholinergic signalling primarily plays an indirect or immunomodulatory role rather than constituting a primary pathogenic mechanism.

Supporting this shared cholinergic involvement, anticholinesterase medications are used therapeutically in both disorders, enhancing acetylcholine availability in the brain in AD and at the neuromuscular junction in MG [15,16]. Moreover, cognitive impairment has been reported in MG patients and is hypothesised to result from central cholinergic disruption or chronic systemic inflammation [1,17,18,19]. Similarly, immune dysregulation, well established in MG [9,11,20], is increasingly recognised in AD, where microglial activation and pro-inflammatory pathways may contribute to neurodegeneration [8,21,22,23]. These shared biological features encompass both central and peripheral immune dysregulation, including gut microbiome-mediated immune signalling along the gut–brain axis, which has been implicated in neuroinflammation in AD [24,25,26]. Together with the reported genetic overlap between AD and gastrointestinal traits [22], this broader immune context raises the possibility of a more complex relationship between AD and MG [1,2,3,17,18,19,27].

Epidemiological studies investigating this potential relationship, however, have produced inconclusive findings. For instance, a recent large retrospective cohort study reported a higher prevalence of AD among older individuals with pre-existing MG: 4.28% of patients with MG over 60 years had an AD diagnosis, compared to 2.82% in the general older population (odds ratio [OR] of 1.5) [1]. This finding is consistent with another recent study, which reported an association between five of twelve autoimmune disorders and AD, with MG showing increased odds of AD (OR 1.49, 95% CI: 1.11–2.00) [3]. Conversely, a large UK record-linkage study reported only a modest association between a broad spectrum of autoimmune diseases and subsequent dementia (rate ratio of 1.06 for AD), and MG was not specifically analysed [27].

Beyond population-level trends, observational studies focused on clinical outcomes suggest that MG may be associated with cognitive dysfunction. For example, a meta-analysis of observational studies reported poorer performance in many domains of cognitive function, including verbal learning and memory, in MG compared to healthy individuals [2]. These findings are largely supported in more recent studies, including systematic reviews and meta-analyses [17,18,19]. While the cognitive impairments reported do not necessarily indicate AD, they may reflect overlapping biological mechanisms. We note, however, that similar cognitive profiles have been described in other autoimmune and inflammatory conditions [28,29], suggesting that such impairments may reflect non-specific effects of systemic immune dysregulation rather than AD-specific pathology. Notably, Mendelian randomisation (MR) analyses examining autoimmune liability (though not specifically MG), and AD risk have generally found little evidence of a strong causal effect, providing limited indirect support for a causal link [30]. Taken together, despite biological plausibility and some epidemiological signals, existing evidence remains inconclusive regarding a specific relationship between AD and MG.

Genome-wide association studies (GWAS) of AD and MG have demonstrated the polygenic architecture of both disorders [20,31,32]. Leveraging these genomic resources, we systematically investigate the extent to which AD and MG share genetic susceptibility and potential causal links. We integrate evidence across genomic scales to distinguish modest genome-wide overlap from locus-specific convergence and to characterise heterogeneous patterns across MG subtypes. We extend this multi-layered framework to incorporate heterogeneity-aware meta-analysis, colocalisation, and expression-informed summary data-based MR (SMR). Collectively, these approaches enable the prioritisation of shared variants, genes, and loci, identify putative expression-mediated regulatory effects, and support biological interpretation through tissue-specific and pathway-based analyses.

## 2. Results

### 2.1. Overview of Study Design

**Figure 1:**
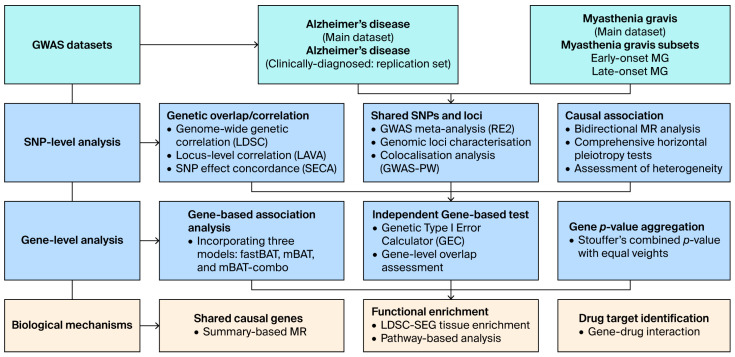
Analysis work flow of the genetic relationship of AD with MG. GEC: genetic error calculator, GWAS: genome-wide association studies, GWAS-PW: GWAS pair-wise, MG: Myasthenia gravis, MR: Mendelian randomisation, SNP: single-nucleotide polymorphism, SECA: SNP effect concordance analysis, LDSC: linkage disequilibrium score regression.

We employed the comprehensive analytical framework summarised in Figure 1. We first estimated genome-wide shared polygenic architecture across AD, MG, early-onset MG (EOMG), and late-onset MG (LOMG) using the linkage disequilibrium score regression (LDSC) method [33]. To evaluate genome-wide concordance in single-nucleotide polymorphism (SNP) effects between AD and MG, we applied SNP Effect Concordance Analysis (SECA) [34]. We also used Local Analysis of [co]Variant Association (LAVA) [35] to identify regions that contribute disproportionately to local correlations between the two disorders. We then performed a heterogeneity-aware cross-trait GWAS meta-analysis to detect potentially shared variants and loci. Across 1703 genomic regions, we applied GWAS-pairwise (GWAS-PW) [36] to determine whether AD and MG share causal variants or instead exhibit trait-specific causal architecture. Using bidirectional MR [37,38,39,40], we investigated potential causal relationships between AD and MG, incorporating multiple MR models, extensive sensitivity tests, and assessments of horizontal pleiotropy and heterogeneity.

Further, we performed a multi-step gene-based analysis to identify putatively shared genetic architecture between AD and MG. Gene-level associations were first assessed using fastBAT, mBAT, and mBAT-combo to capture aggregated SNP effects [41], and these results were subsequently integrated across traits using Stouffer’s method to prioritise putatively shared genes. In parallel, independent gene-based analysis was conducted by applying GATES to identify the most significant SNP per gene, followed by Genetic Type-1 Error Calculator (GEC)-based modelling of inter-gene independence to estimate the effective number of independent genes [42]. The resulting GEC-derived outputs were then used to quantify gene-level overlap between AD and MG through enrichment analyses across predefined significance thresholds, enabling robust evaluation of shared genetic signal beyond variant-level associations. We applied SMR with the Heterogeneity InDependent Instruments (HEIDI) test to identify putative causal genes and distinguish pleiotropy from linkage. We then performed pathway enrichment analyses to highlight the biological processes implicated by these gene sets [43,44]. Finally, we assessed therapeutic relevance by mapping prioritised genes to known drug targets using gene–drug interaction databases [45]. All analyses in this study were conducted using a consistent genome build (GRCh37/hg19), and no additional liftover procedures were required. Supplementary Table S1 and the Methods section provide additional details on the datasets and analytical procedures used.

### 2.2. Genome-Wide Genetic Correlation of AD with MG

**Figure 2:**
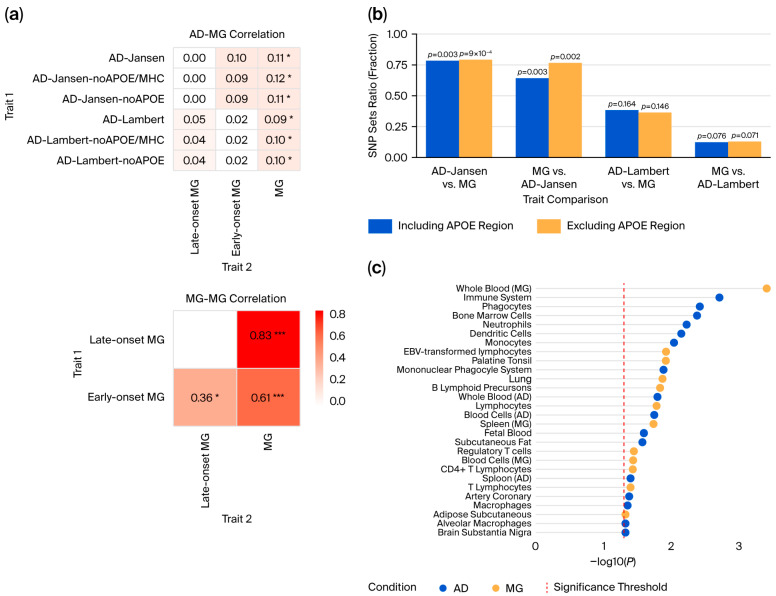
Results of genetic correlation, SNP effect concordance, and enrichment of SNP heritability of AD with MG. AD: Alzheimer’s disease, MG: Myasthenia gravis, *p*: permuted *p*-value, MHC: Major Histocompatibility Complex. (**a**): Genetic correlations between AD and MG and MG subtypes. The heatmap visualises pairwise genetic correlation estimates between AD (from Jansen and Lambert GWAS) and MG (overall, early-onset, and late-onset subtypes). Rows and columns represent trait pairs; cell colours reflect the direction and magnitude of rg, with values annotated inside each tile. Asterisks indicate significance: *p*  <  0.05 (*), and < 0.001 (***). Where sample overlap appeared negligible (gencov intercept ≈ 0), the genetic covariance intercept was constrained; unconstrained estimates were Gencov (Early-onset vs. MG) = 0.48 (SE 0.0091) and (Late-onset vs. MG) = 0.23 (SE 0.012). (**b**): SECA tested directional concordance of SNP effects between AD and MG using overlapping independent SNPs from two AD GWAS ([31,32]) and MG, in both AD → MG and MG → AD directions, with and without the APOE region. Each bar in the analysis represented the fraction of SNPs with concordant positive effects (OR > 1; P1, P2 < 0.05); low permuted *p*-values (e.g., 0.003, 0.0009) indicated more concordant SNP subsets than expected by chance. (**c**): Stratified LD score regression highlighted tissue-specific SNP-heritability enrichment for both traits, with immune-related tissues. The dotted line represents cell types showing nominally significant enrichment (*p* < 0.05), suggesting immune contributions to shared genetic architecture.

We observed a nominally significant positive genetic correlation between AD and MG (rg = 0.11, *p* = 2.07 × 10^−2^), which persists after excluding the *APOE* region (rg = 0.11, *p* = 3.62 × 10^−2^) and both the *APOE* and the MHC regions (rg = 0.12, *p* = 2.18 × 10^−2^, Figure 2a, and Supplementary Table S2). We found a similar pattern using the clinically diagnosed AD GWAS, where correlations were nominally significant (full genome: rg = 0.09, *p* = 3.85 × 10^−2^; *APOE*-removed: rg = 0.10, *p* = 4.01 × 10^−2^; *APOE*+MHC-removed: rg = 0.10, *p* = 4.01 × 10^−2^). After adjusting for multiple testing across all LDSC comparisons (Bonferroni threshold = 0.05/5 = 0.01), none of the AD–MG correlations survived, indicating that these signals are noteworthy but should be interpreted as nominal evidence of shared polygenic risk. In contrast, correlations between MG and its subtypes were strong and highly significant, surpassing multiple-testing thresholds (LOMG vs. MG: rg = 0.83, *p* = 2.20 × 10^−6^; EOMG vs. MG: rg = 0.61, *p* = 9.83 × 10^−9^). The correlation between EOMG and LOMG was weaker and only nominally significant (rg = 0.36, *p* = 2.40 × 10^−2^). Across analyses of MG subtypes, AD showed consistently small and non-significant correlations in both the discovery and replication datasets, regardless of whether the APOE or MHC regions were excluded (Figure 2a and Supplementary Table S2).

### 2.3. SNP-Effect Concordance Analysis of AD with MG

In the analysis comparing AD with MG (Figure 2b, and Supplementary Table S2), 113 of 144 subsets showed concordant effect directions (Fisher’s test OR ≥ 1, *p* < 0.05), more than expected by chance (empirical P_permuted_ = 0.003). In the reverse analysis (MG as dataset 1, AD as dataset 2), 92 subsets were significant (P_permuted_ = 0.003, Supplementary Table S2). These findings were consistent in the analysis excluding the APOE region and indicate that some of the SNPs strongly associated with AD also tend to influence MG, and vice versa, supporting SNP-level concordance between the two disorders. In contrast, when using the clinically diagnosed AD dataset, only 55 of 144 subsets showed concordant effect directions (Fisher’s test OR ≥ 1, *p* < 0.05, Figure 2b and Supplementary Table S2), which was not more than chance expectation (P_permuted_ = 0.164). The reverse comparison (MG as dataset 1, AD as dataset 2) identified 17 significant subsets (P_permuted_ = 0.076). These results were consistent after excluding the APOE region (Figure 2b). Together, significant effect concordance between AD and MG was not replicated using the clinically diagnosed AD dataset, possibly due to the smaller sample size of the AD GWAS. SECA findings should be interpreted in the context of the full analytical framework rather than as a stand-alone test of shared genetic architecture.

### 2.4. Tissue- and Cell-Type-Specific Heritability Enrichment

Partitioned heritability analysis using Stratified LD score regression (S-LDSC) with specifically expressed gene (SEG) annotations showed significant enrichment of SNP heritability in immune-related tissues for both AD and MG, consistent with shared immunogenetic contributions (Figure 2c, Supplementary Table S3). For AD, enrichment was concentrated in myeloid-derived immune cells, including the immune system, phagocytes, bone-marrow-derived cells, neutrophils, monocytes, and dendritic cells. Additional signals in whole blood, macrophages, and the brain substantia nigra further support the contribution of immune dysregulation and neuroinflammation to AD pathogenesis.

For MG, the strongest enrichment occurred in whole blood, followed by EBV-transformed lymphocytes, palatine tonsil, lung, spleen, and multiple B- and T-lymphocyte subsets. Overall, these findings highlight immune-tissue enrichment for both disorders, with AD showing stronger myeloid-derived (innate immune) signatures and MG enriched in lymphoid and adaptive immune populations, supporting shared yet disease-specific immunogenetic mechanisms.

### 2.5. Local Genetic Correlations of AD with MG and MG Sub-Types

Findings from our local genetic correlation analysis, using the LAVA approach [35], provide locus-level insights into the relationship of AD with MG (and MG subtypes).

#### 2.5.1. Multi-Trait Locus-Level Genetic Correlation of AD with MG and MG-Subtypes

In the multi-trait LAVA analysis, which jointly models AD, MG, and its subtypes (EOMG and LOMG), we identified multiple genomic loci showing significant or suggestive local genetic correlations, primarily concentrated within the MHC region, as well as additional loci across other chromosomes (chr, Table 1). For AD–MG, five loci exhibited significant or suggestive local correlations. The most robust, Bonferroni-corrected (*p* < 3.42 × 10^−4^, accounting for 146 analyses) signal was at chr6:32.59–32.63 Mb (locus 965; ρ = 0.35, *p* = 9.2 × 10^−5^) within the classical MHC region. Two closely spaced MHC loci also showed suggestive (3.42 × 10^−4^ < *p* < 0.05) positive correlations (locus 964: chr6: 32.54–32.59 Mb, ρ = 0.33, *p* = 8.0 × 10^−4^; locus 966: chr6: 32.63–32.68 Mb, ρ = 0.24, *p* = 3.6 × 10^−2^), indicating an extended association across this region. Another locus bordering the MHC (chr6: 27.26–28.67 Mb; locus 952; ρ = 0.23, *p* = 3.9 × 10^−2^) also showed a positive correlation. Importantly, we detected a suggestive signal outside the MHC region at chr3: 47.59–50.39 Mb (locus 464; ρ = 0.75, *p* = 3.3 × 10^−3^), suggesting that genetic influences between AD and MG extend beyond the MHC locus (Table 1). Although the chromosome 3 finding was formally classified as suggestive, it remains notable due to the relatively large local genetic correlation estimate (ρ = 0.75). Moreover, the confidence interval extends to 1.00, which—within the LAVA framework—is consistent with the possibility of near-complete sharing of local genetic effects between AD and MG, even though it does not survive Bonferroni correction.

For AD–LOMG, we identified one significant and several additional loci with suggestive local correlations (Table 1). The strongest signal was at chr6:32.63–32.68 Mb (locus 966; ρ = 0.51, *p* = 3.0 × 10^−5^) within the MHC region, indicating a significant positive correlation between AD and LOMG. Other loci within or near the MHC region also showed positive correlations, including chr6:31.25–31.32 Mb (locus 959; ρ = 0.55, *p* = 3.9 × 10^−2^), and chr6:32.21–32.45 Mb (locus 962; ρ = 0.30, *p* = 4.9 × 10^−2^). We also detected an association at chr16:53.4–54.9 Mb (locus 2135; ρ = 0.54, *p* = 8.9 × 10^−4^) outside the MHC region. These findings indicate a significant correlation between AD and LOMG that is not limited to the MHC region. We observed both positive and negative local genetic correlations between AD and LOMG, suggesting that the traits share a heterogeneous genetic architecture. Loci showing positive correlation suggest concordant genetic effects, while those showing negative correlation (e.g., chr 18), reflect opposing, trait-divergent effects, which may be consistent with antagonistic pleiotropy.

For AD–EOMG, no loci reached Bonferroni-corrected significance (Table 1); however, we identified several suggestive positive correlations, all within the MHC region. The strongest signal was at chr6:32.59–32.63 Mb (locus 965; ρ = 0.24, *p* = 1.99 × 10^−3^), followed by nearby regions at chr6:32.54–32.59 Mb (locus 964; ρ = 0.21, *p* = 2.5 × 10^−2^), chr6:30.07–30.72 Mb (locus 956; ρ = 0.29, *p* = 3.0 × 10^−2^), and chr6:25.68–26.40 Mb (locus 950; ρ = 0.29, *p* = 1.3 × 10^−2^). All loci demonstrated moderate positive correlations, suggesting concordant local genetic architecture between AD and EOMG, primarily within the extended MHC region.

#### 2.5.2. Pairwise Locus-Level Genetic Correlation of AD with MG and MG-Subtypes

Pairwise local genetic correlation analyses (AD–MG, AD–LOMG, and AD–EOMG) maximise the number of shared SNPs across datasets, facilitating finer resolution of subtype-specific effects. Positive local genetic correlation indicates concordant regional genetic effects between traits, whereas negative correlation indicates opposing local genetic effects within a genomic region. We identified several loci showing significant or suggestive local correlation (Table 2). In the AD–MG model, two loci reached significance after correction for 35 tests (*p* < 1.43 × 10^−3^). The strongest signal mapped to chr16:53.39–54.87 Mb (locus 2135; ρ = 0.54, *p* = 1.27 × 10^−4^), reflecting a robust positive correlation outside the MHC, with evidence consistent with near complete sharing. A second significant correlation was in the MHC class II region at chr6: 32.59–32.63 Mb (locus 965; ρ = 0.19, *p* = 1.58 × 10^−3^). Two additional loci showed suggestive associations (*p* < 0.05): one in the extended MHC region (chr6: 29.53–29.83 Mb; locus 954; ρ = 0.35, *p* = 2.83 × 10^−2^) and one on chr18 (chr18: 20.01–21.62 Mb; locus 2255; ρ = –0.23, *p* = 3.55 × 10^−2^). These results indicate limited but meaningful local overlap between AD and MG, involving loci within and outside the MHC.

In the AD–LOMG analyses, six loci were significant (*p* < 2.08 × 10^−3^ across 24 tests), including some within the MHC. The strongest correlation was a highly significant positive effect at chr1:113.4–114.7 Mb (locus 100; ρ = 0.90, *p* = 8.24 × 10^−7^), followed by others in MHC regions (loci: 964, 965, and 959). Several suggestive loci were distributed across both MHC (e.g., loci 958, 960, and 955) and non-MHC regions, including chrs15 and 18. This finding indicates a mixture of concordant and opposing local effects between AD and LOMG, involving both immune and non-immune loci.

Pairwise AD–EOMG analyses revealed multiple significant or suggestive local correlations, largely confined to the MHC region (*p* < 2.08 × 10^−3^ for 24 tests). The strongest effect was a negative correlation at chr6:32.63–32.68 Mb (locus 966; ρ = –0.23, *p* = 1.26 × 10^−5^). Nearby loci demonstrated positive correlations, including chr6:33.19–33.86 Mb (locus 969; ρ = 0.46, *p* = 8.43 × 10^−4^). Additional suggestive signals spanned both positive (e.g., chr6: 30.07 Mb; locus 956; ρ = 0.29) and negative (e.g., chr6: 32.45–32.54 Mb; locus 963; ρ = −0.49) directions, together highlighting fine-scale heterogeneity within the MHC. A single non-MHC suggestive association was at chr11: 75.4–76.5 Mb (locus 1682; ρ = −0.34, *p* = 3.56 × 10^−2^). These findings indicate that the AD–EOMG relationship is primarily driven by MHC substructure, with both concordant and divergent local effects.

### 2.6. Genome-Wide Significant SNPs and Loci Shared by AD and MG in GWAS Meta-Analysis

We conducted a heterogeneity-aware cross-trait GWAS meta-analysis of AD and MG using METASOFT’s RE2 model, the Binary effect (BE) *p*-value, and the *m*-value framework [46]. Based on the meta-analysis results (Table 3), we classified findings into three groups: (1) Genome-Wide Significant (GWS) SNPs and loci that reached significance only after the meta-analysis (putatively novel loci in the context of our data), with support from BE *p*-value and *m*-value, (2) SNPs/loci previously GWS for AD that also showed evidence of association with MG (P_MG_ < 0.05), supported by the meta-analysis parameters, and (3) SNPs/loci previously GWS for MG that demonstrated association with AD (P_AD_ < 0.05), with corroboration from the meta-analysis metrics (BE *p*-value and *m*-value).

In the first category, a total of 11 independent SNPs across two genomic regions on chr6 and 16 reached GWS (*p* < 5 × 10^−8^), despite being sub-threshold in AD and MG GWAS (Table 3). This finding was supported by both the BE *p*-values and the *m*-values. The BE *p*-value tests for an effect in at least one study, with low values indicating SNP association with one or both traits. M-values ≥ 0.9 in AD and MG suggest a high probability of shared association. This criterion was met by all identified SNPs in this group except rs9270505, which had an *m*-value < 0.9 for AD. This *m*-value does not undermine potential association, as multiple independent SNPs with consistent strong evidence support the locus (in our results). The second locus (rs889555, chr16) also reached GWS, with a high *m*-value for AD but an ambiguous *m*-value (0.22) for MG. This region was identified in our gene-based analyses of both traits. Notably, a search in the GWAS Catalog (11 July 2025) confirms this locus for AD but is putatively novel for MG (Supplementary Table S4).

In the second category, we identified 12 independent SNPs across six loci GWS in the AD GWAS and showed at least nominal significance in MG (Table 3). These SNPs were also GWS in the meta-analysis and largely supported by both BE *p*-values and *m*-values (suggesting the association was not predominantly driven by one of the traits). All SNPs in this group had *m*-values > 0.9 for AD, supporting their strong association. For MG, most SNPs also had *m*-values greater than or close to 0.9 (for example, >0.8), supporting likely shared effects. Notable loci in this group include variants on chr7 (rs6979218, rs35251323, rs62472729) and chr19 (rs3752241, rs1871046, rs143668237, rs874744), the latter being a region well-established in AD. Based on a GWAS Catalog search (11 July 2025), the independent SNP rs13201473 (chr6) has not been previously reported for MG, suggesting a putatively novel association. Similarly, the chr7 loci have been linked to other autoimmune conditions but not MG. We also found no prior evidence connecting rs59735493 (chr16) or the chr19 variants to MG, supporting their putative novelty (Supplementary Table S5).

In the third category, we identified two independent SNPs, rs9271163 and rs9271548, both in the MHC region. These SNPs were GWS in the MG GWAS and reached GWS in the meta-analysis, supported by the BE *p*-values (Table 3). Although their *m*-values for AD were in the uncertain range (0.22–0.26), both SNPs had already reached GWS in the AD GWAS before the meta-analysis, supporting involvement in both disorders. More comprehensive results for these findings are provided in Supplementary Table S6.

### 2.7. Inconclusive Causal Effect of MG on AD

A summary of our MR analysis framework and an overview of its underlying assumptions are presented in Supplementary Figure S1. We considered our findings significant at *p* < 0.025 (0.05/2, Bonferroni adjustment for assessing two traits), and nominally significant at *p* < 0.05. First, we found no evidence of a causal influence of AD on MG. The primary model (IVW) produced an odds ratio (OR) of 0.83 (95% CI: 0.59–1.16, *p* = 0.28), and similar null results were obtained with the weighted median (OR = 0.83, 95% CI: 0.51–1.36, *p* = 0.46) and MR Egger (OR = 0.86, 95% CI: 0.54–1.38, *p* = 0.54) methods. The MR-PRESSO result supported these findings (OR = 0.83, 95% CI: 0.60–1.14, *p* = 0.25) [Supplementary Table S7]. Parallel analyses using another AD GWAS (for potential [partial] replication) yielded concordant null effects (IVW: OR = 1.02, 95% CI: 0.96–1.06, *p* = 0.85), suggesting no evidence that AD exerts a causal influence on MG (Supplementary Table S7).

However, when we examined the effect of MG on AD, the findings were slightly different. With AD as the outcome, the IVW model revealed a weak yet statistically significant association for genetically predicted MG liability (i.e., per unit increase in log-odds of MG liability) (OR = 1.013, 95% CI: 1.004–1.021, *p* = 2.67 × 10^−3^) [Supplementary Table S7]. This result was close to marginal but supported by the weighted median method (OR = 1.012, 95% CI: 1.001–1.023, *p* = 1.44 × 10^−2^) and MR-PRESSO (Raw; OR = 1.013, 95% CI: 1.007–1.018, *p* = 1.50 × 10^−3^). All instrumental variables (IVs) showed F-statistics well above 10 (approximately 30.0 to 151), indicating that weak instrument bias is unlikely (Supplementary Table S7). The MR-Egger intercept test for this analysis revealed no evidence of horizontal pleiotropy, as the intercept remained close to zero with no significant deviation (Supplementary Table S7). Similarly, we did not detect significant heterogeneity (Supplementary Table S7). However, (partial) replication testing revealed no evidence of a significant influence of MG on AD: IVW (OR = 1.04, 95% CI: 0.95–1.15, *p* = 0.37).

We note that the analysis using the MG as the exposure variable had limited IVs (fewer than 10); hence, we pursued an exploratory investigation by conducting analyses using IVs for MG selected at a genome-wide suggestive level (*p* < 1 × 10^−6^). We observed a borderline significant association for the putative effect of genetically predicted MG liability on AD in the weighted median model (OR = 1.01, 95% CI: 1.00–1.02, *p* = 0.049) [Supplementary Table S7], which is not convincing. This result was not consistent across other models, including the IVW, MR Egger, and MR-PRESSO, and no association was observed for clinically diagnosed AD GWAS (Supplementary Table S7).

We performed further assessment using the bi-directional Generalised Summary-data MR approach, in which IVs were selected at both genome-wide (*p* < 5 × 10^−8^) and suggestive (*p* < 1× 10^−6^) thresholds (Supplementary Table S7). When MG was the exposure and AD the outcome, we observed a weak but significant association between genetically predicted MG liability and AD risk. At the genome-wide threshold, the effect estimate was OR =1.011 (95% CI: 1.003–1.019), *p* = 0.01; IVs = 10), with no evidence of horizontal pleiotropy (HEIDI test *p* = 0.98). Using suggestive IVs to increase instrument count, the association tended to be significant, but the effect was weaker: OR =1.008 (95% CI: 1.002–1.014), *p* = 0.0137; IVs = 22), with HEIDI *p* = 0.82 (Supplementary Table S7). Overall, our MR analysis does not support a causal effect of AD on MG. However, the putative influence of MG on AD cannot be overlooked, with sensitivity analyses suggesting that horizontal pleiotropy is unlikely to explain the observed marginal associations. This finding warrants further investigation as more robust MG GWAS data become available.

### 2.8. Shared Loci for AD and MG in Colocalisation Analysis

GWAS-PW investigates shared genetic loci between AD and MG. The approach aims to identify loci likely to harbour either a single pleiotropic variant that causally influences both traits (evaluated by posterior probability of association [PPA]3) or two independent causal variants that contribute separately to each trait (PPA4). We report loci with strong support (>90% PPA, indicating high confidence) and moderate-high support (>50% PPA) for both PPA3 and PPA4 scenarios. Our analysis identified several loci with significant evidence for independent causal variants contributing separately to each trait (PPA4). Key findings are summarised in Supplementary Figure S2 and Supplementary Table S8.

With a PPA4 of 1.00, the locus at chr6: 31,571,971–32,682,443 (hg19) shows the strongest support for a shared regional association between AD and MG driven by distinct causal variants (Supplementary Table S8). The corresponding logBF4 of 52.84 further supports this model. The locus at chr4: 10,699,425–12,322,014 (hg19) also shows strong posterior support for distinct causal variants (PPA4 = 0.88). Other loci, including chr19: 610,770–2,098,063 (PPA4 = 0.63) and chr6: 32,682,664–33,236,175 (hg19, PPA4 = 0.56), show moderate support for shared regional association associated by distinct causal variants.

By contrast, chr16: 29,036,613–31,379,355 (hg19) showed moderate support for a shared causal variant (PPA3 > 0.60), suggesting putative colocalisation at this locus. Interpretation of PPA3 versus PPA4 should remain cautious in regions of complex LD or multiple causal variants, where GWAS-PW may have limited power to fully resolve the underlying model.

### 2.9. Independent Genes and Gene-Level Overlap of AD with MG

We estimated the effective number of independent genes associated with AD and MG across three significance thresholds (P_gene_ < 0.1, P_gene_ < 0.05, and P_gene_ < 0.01; Table 4) using the GEC approach. Gene-based association analyses were performed using the GATES method [47], implemented within the Fast Association Tests (FAST) framework [48], which is particularly suited for independent gene-level testing (details in Methods). To determine whether AD and MG shared more associated genes than expected by chance, we compared the proportion of observed overlapping independent genes with the expected proportion under the null hypothesis.

Our results consistently demonstrated that the observed overlap exceeded expectations at all thresholds. Binomial tests confirmed the association, providing strong evidence of a gene-level overlap between AD and MG (Table 4). For example, at P_gene_< 0.05, the observed proportion of overlapping genes (14.0%) was significantly higher than the expected proportion (8.8%) (P_binomial-test_ = 4.33 × 10^−12^). This trend persisted at a more stringent threshold (P_gene_ < 0.01, P_binomial-test_ = 1.49 × 10^−9^) and the slightly less stringent level (P_gene_ < 0.1, P_binomial-test_ = 1.66 × 10^−15^).

#### 2.9.1. Cross-Trait Genome-Wide Significant Gene Overlap Between AD and MG

We applied Stouffer’s Z-score method with equal weights to combine gene-based *p*-values from AD and MG. Briefly, this approach transforms individual *p*-values into Z-scores, averages them, and derives a combined *p*-value that reflects joint evidence of association across traits. Unlike Fisher’s method, which can be disproportionately influenced by a single extremely small *p*-value, Stouffer’s method reduces such influence by averaging, making it more robust to outliers. This feature enhances the method’s utility, particularly when modest associations are observed in both traits.

First, we assessed the overlap of genes that reached GWS in gene-based analyses for AD (P_gene-AD_ < 2.67 × 10^−6^) and MG (P_gene-MG_ < 2.67 × 10^−6^). Eight genes met this criterion for both disorders and are presented in Table 5. All identified genes are located within the extended MHC region, including *HLA-DQB1*, *BTNL2*, *TSBP1*, *HLA-DRA*, *HLA-DQA1*, *HLA-DRB1*, *HLA-DQA2*, and *HLA-DQB2*. For each of these genes, we observed strong associations in both traits independently, as indicated by their top SNPs and gene-level *p*-values (P_mBATcombo_). To assess the joint genetic signal across traits, we applied Stouffer’s Z-score method with equal weighting. All eight genes showed extremely significant combined Z-scores (e.g., *HLA-DQB1*, Z = −11.89, *p* = 6.45 × 10^−33^), reinforcing their potentially shared relevance in AD and MG. These findings suggest convergent immune-related association signals between AD and MG, particularly within the extended MHC class II region.

In the second step of our analysis, we identified genes that were GWS in the AD gene-based analysis (P_gene-AD_ < 2.67 × 10^−6^), but only showed sub-threshold associations in MG (0.001 < P_gene-MG_ > 2.67 × 10^−6^). Although these genes did not individually meet GWS in MG, we assessed whether the joint evidence from both traits would support putative shared genetic involvement. Several genes, most notably *ZNF668*, *CFAP119*, *ARHGAP45*, *POLR2E*, and *ABCA7*, reached GWS after the combined analysis (e.g., *POLR2E*, Z = −7.30, *p* = 1.43 × 10^−13^). Many of these genes, particularly those on chr19 (e.g., *ARHGAP45*, *POLR2E*, *ABCA7*, *CNN2*, *GPX4*), clustered in a region previously implicated in AD susceptibility. These findings highlight potential shared genetics between AD and MG that may not be detectable when analysing either disorder in isolation.

In the third step, we focused on genes that reached GWS in the MG gene-based test (P_gene-MG_ < 2.67 × 10^−6^), but not in AD (0.001 <P_gene-AD_ > 2.67 × 10^−6^). We uncovered joint associations for several genes, including *ABHD16A*, *C6orf15*, *NOTCH4*, and *CDSN*, which surpassed GWS thresholds (Supplementary Table S9). Many of these genes are clustered within the MHC region. Genes located within MHC-associated loci were interpreted primarily in the context of regional genomic localisation and should not necessarily be considered functionally immune-specific, given the extensive pleiotropy and LD structure of the region.

#### 2.9.2. Genes Reaching Genome-Wide Significance for AD and MG in the Combined *p*-Value Analysis

**Figure 3:**
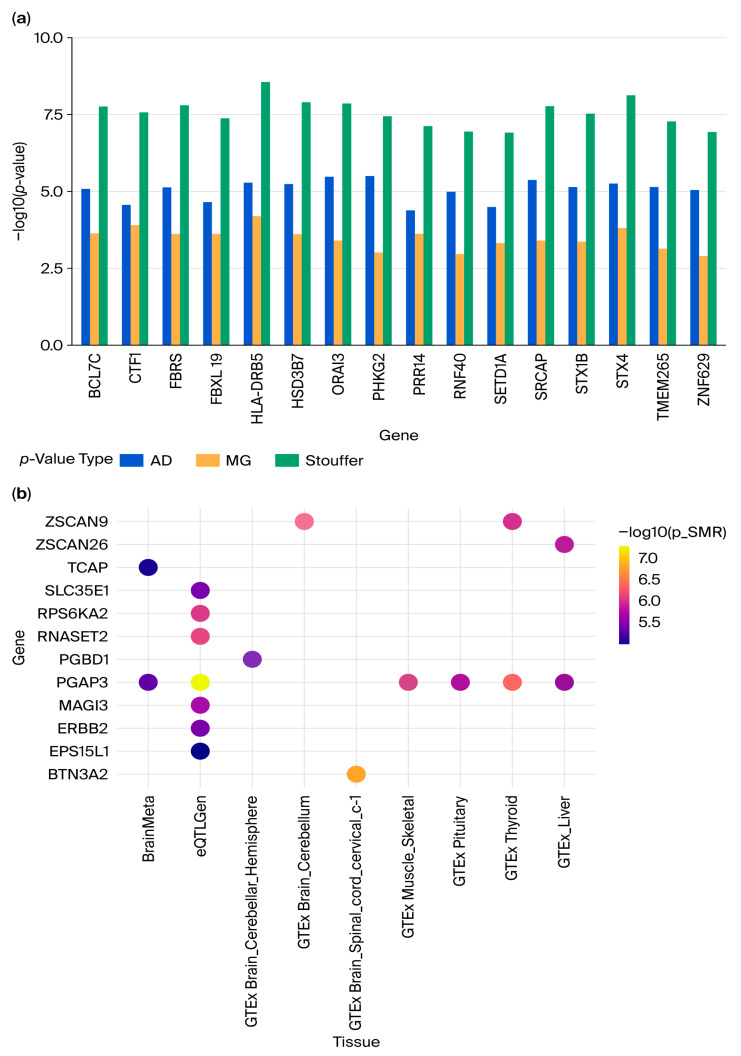
Genes reaching GWS for AD and MG, and MG putative causal genes. (**a**) Bar plot of gene-level associations for AD, MG, and cross-trait combined *p*-value using the Stouffer method. The −log_10_ (*p*-value) is plotted for each gene across the three *p*-value types (P_AD_, P_MG_, P_Stouffer_). Genes with stronger association signals (lower *p*-values) appear taller, and colour represents the *p*-value type. (**b**) Bubble plot displaying genes with putative causal associations with MG across different tissues, based on gene expression SMR analysis. Each bubble represents a gene–tissue pair, with bubble size and colour corresponding to −log_10_(pSMR), indicating the strength of association. AD: Alzheimer’s disease, GWS: genome-wide significant, MG: myasthenia gravis, SMR: summary data-based Mendelian randomisation.

We conducted a further assessment to identify genes that reached GWS in the combined *p*-value analysis (P_Stouffer’s-combined-analysis_ < 2.67 × 10^−6^) but did not meet GWS thresholds in either AD or MG individually (P_gene-AD_ > 2.67 × 10^−6^; P_gene-MG_> 2.67 × 10^−6^). To reduce the likelihood that such signals were driven predominantly by a single trait, we further required evidence of at least moderate association in both traits (P_gene-AD_ < 0.001 and P_gene-MG_ < 0.001). This approach aimed to identify putatively novel loci supported by moderate associations in both traits. Our results revealed several such genes that surpassed the GWS threshold in the combined analysis, despite being sub-threshold in the trait-specific analyses (Figure 3a and Supplementary Table S10). The strongest signal was observed for *HLA-DRB5* (*p* = 2.83 × 10^−9^), supported by nominally significant gene-based *p*-values in both AD and MG (P_mBAT-combo_: 5.16 × 10^−6^ and 6.42 × 10^−5^, respectively). Notably, we identified a cluster of genes on chr16, including *PRR14*, *FBRS*, *SRCAP*, *TMEM265*, *PHKG2*, *RNF40*, *ZNF629*, *BCL7C*, *CTF1*, *FBXL19*, *ORAI3*, *SETD1A*, *HSD3B7*, *STX1B*, and *STX4*, all of which surpassed GWS following the combined analysis.

### 2.10. Cross-Tissue SMR Identifies Putative Causal Genes in MG and AD

Using the gene expression-based SMR, we first identified several putative causal genes for MG that surpassed the Bonferroni-corrected significance (GWS, *p* < 9.99 × 10^−6^, Figure 3b and Supplementary Table S11). Notably, *PGAP3* emerged consistently across multiple tissues, including whole blood, thyroid, skeletal muscle, pituitary, liver, and brain, supporting strong cross-tissue evidence of its potential role in MG (pSMR range: 5.43 × 10^−8^ to 4.72 × 10^−6^). Similarly, *ZSCAN9* was significant in both the brain cerebellum and thyroid, and *BTN3A2* showed a significant association in the spinal cord. Additional genes demonstrating significant associations include *RNASET2*, *RPS6KA2*, *ZSCAN26*, *MAGI3*, *SLC35E1*, *PGBD1*, *ERBB2*, *TCAP*, and *EPS15L1*, identified in at least one tissue (Figure 3b, and Supplementary Table S11). All associations passed the HEIDI test (pHEIDI > 0.01, nSNP HEIDI > 20), hence, they are unlikely to be due to linkage. Except *MAGI3*, all identified genes are putatively novel for MG, based on the GWAS Catalog (accessed 5 June 2025). Notably, *RPS6KA2* and *PGBD1* have been associated with AD or dementia-related traits, lending support to a potential shared genetic basis between MG and AD. The most frequently implicated tissue was whole blood (associations for seven genes), followed by the thyroid, liver, and various brain regions.

**Figure 4:**
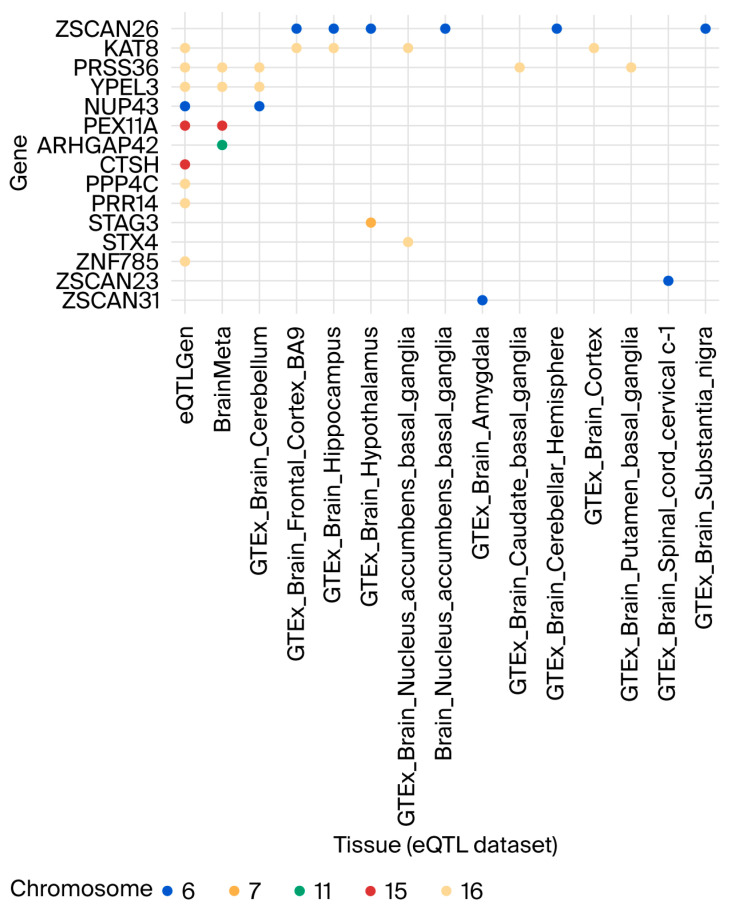
Putatively shared causal genes of AD with MG identified by the SMR analysis. The horizontal-axis lists the individual eQTL reference datasets used in the SMR analysis, including whole-blood and brain meta-analysis resources (eQTLGen, BrainMeta) as well as tissue-specific brain eQTL datasets from GTEx. The vertical-axis shows genes prioritised by SMR as putatively mediating shared genetic associations between AD and MG. Each point represents a significant SMR association for a given gene in a specific eQTL dataset, indicating evidence that genetically regulated expression of that gene in the corresponding tissue may contribute to the observed cross-trait association. Point colours denote the chromosome on which each gene is located.

Second, we identified a set of putatively shared causal genes for AD and MG, supported by multi-tissue eQTL data (Figure 4). All genes reported passed FDR correction and met additional stringency criteria, including a HEIDI test *p*-value > 0.01 and nHEIDI > 20, indicating that the associations are unlikely due to linkage (Supplementary Table S12). Fifteen unique genes were significantly associated with both AD and MG across at least one brain region or eQTL dataset.

These genes are in five chrs (chrs6, 7, 11, 15, and 16), with clustering on chrs6 and 16. Several genes, including *ZSCAN26*, *ZSCAN23*, and *ZSCAN31* on chr6, were identified across multiple GTEx brain regions, such as the frontal cortex, hippocampus, cerebellum, and substantia nigra. This finding is consistent with shared, broadly acting regulatory influences across AD-relevant brain tissues. Similarly, chr16 harboured several shared genes, including *KAT8*, *PRSS36*, *YPEL3*, *PPP4C*, and *STX4*, many of which were supported by both GTEx and other eQTL datasets such as eQTLGen and BrainMeta. Notably, *KAT8* and *PRSS36* were each associated with multiple brain regions, including the nucleus accumbens, putamen, and hippocampus, consistent with cross-tissue regulatory signals in both neural and peripheral contexts. Additional shared genes included *NUP43* (chr6), *PEX11A* (chr15), *ARHGAP42* (chr11), and *STAG3* (chr7), showing further evidence of overlapping immune–neuronal-related genes between the two disorders.

### 2.11. Significantly Enriched Biological Pathways for AD and MG

Pathway enrichment analysis of genes jointly implicated in AD and MG, whose loci are supported by at least two independent analytical methods (Supplementary Table S13), revealed consistent overrepresentation of immune-related processes. The pathways were across Gene Ontology (GO), Kyoto Encyclopedia of Genes and Genomes (KEGG), Reactome (REAC), and WikiPathways (WP). These converged on three dominant biological themes: MHC class II antigen presentation, infection and autoimmune-related pathways, and T cell receptor (TCR) and interferon signalling. GO enrichment highlighted strong signals for MHC class II functions, including ‘MHC class II protein complex binding’ (FDR = 5.2 × 10^−8^) and ‘antigen processing and presentation of exogenous peptide antigen via MHC class II’ (FDR = 5.96 × 10^−7^), largely driven by *HLA-DRA*, *HLA-DQA1*, *HLA-DRB1*, *HLA-DQB1/2*, and *HLA-DRB5* (Supplementary Table S14). KEGG analysis supports this immune signature, showing enrichment in autoimmune disease pathways (e.g., type I diabetes, rheumatoid arthritis, systemic lupus erythematosus) as well as pathways related to immune responses in infectious contexts (e.g., Epstein–Barr virus, tuberculosis, SARS-CoV-2) (Supplementary Table S15). Together, these overlapping pathways are consistent with a convergent immune-related genetic architecture shared by AD and MG.

REACT and WikiPathways annotations further implicated T-cell activation and interferon signalling (Supplementary Table S16). Notable enrichments included ‘ZAP-70 translocation to the immunological synapse’ (FDR = 5.98 × 10^−8^), ‘interferon gamma signalling’, and ‘co-inhibition by PD-1’, highlighting potential convergence on altered T-cell signalling and interferon-related immune pathways in AD and MG. Together, these results support a shared neuroimmune axis involving antigen presentation and adaptive immune regulation, with engagement of immune pathways that are also commonly activated in inflammatory and infectious contexts.

### 2.12. Gene–Drug Interactions

Gene–drug interaction analysis of AD–MG shared genes revealed several actionable targets with regulatory-approved compounds, suggesting potential opportunities for drug repurposing or safety monitoring (Supplementary Table S17). *VKORC1*, a gene central to vitamin K metabolism, was linked to multiple anticoagulants, warfarin, acenocoumarol, phenprocoumon, and dicumarol, with high interaction scores (up to 6.96). Notably, investigational compounds, fluindione and tecarfarin, showed even stronger predicted interactions (score: 15.66), highlighting *VKORC1*’s pharmacogenomic relevance. Several HLA class II genes, *HLA-DRB1*, *HLA-DQB1*, *HLA-DQA1*, and *HLA-DRA*, were associated with approved immunomodulatory and anti-inflammatory agents, including infliximab, adalimumab, tocilizumab, azathioprine, and interferon beta. Other shared genes, such as *STX1B* and *STX4*, involved in vesicle trafficking, showed high interaction scores with warfarin and phenprocoumon, while *NOTCH4* was linked to approved and experimental Notch pathway inhibitors (e.g., nirogacestat, MK0752).

## 3. Discussion

We present a comprehensive investigation of the genetic relationship between AD and MG across genome-wide, gene-level, and locus-specific analyses, complemented by expression-, pathway-, and causal-inference approaches. We identified a nominally significant genome-wide genetic correlation that persisted after excluding the APOE and MHC regions and was replicated using clinically diagnosed AD data. These results indicate that the estimated genetic correlation is not driven solely by canonical loci but may reflect a modest, diffuse shared polygenic component. At the gene level, we observed robust and greater-than-expected overlap, while SECA detected significant SNP-level concordance in the primary GWAS but not in the smaller clinically diagnosed AD dataset, most likely reflecting reduced statistical power or sensitivity to association strength in the latter. While the inclusion of AD-by-proxy cases in the primary GWAS may introduce broader signals, potentially contributing to this finding, we consider this a secondary factor relative to the substantial difference in sample size, especially given that SECA is sensitive to sample size [34]. To our knowledge, the current study provides the first integrated investigation of shared susceptibility between AD and MG, offering genetic evidence for a modest shared component. Notably, the modest genome-wide correlation, despite robust gene-level overlap, likely reflects the fact that gene-based approaches capture aggregated signals, whereas genome-wide correlation estimates are more sensitive to heterogeneous or opposing effects across loci. This premise potentially explains the inconsistencies in previous epidemiological findings and refines earlier observational reports that suggested links between AD and MG [1,2,17,19,27].

Local genetic correlation analysis provides higher-resolution insight by identifying loci that disproportionately influence the relationship between AD and MG, without implying that weaker (non-detectable) shared effects are absent elsewhere in the genome [35]. Consistent with this view, the AD–MG local correlations were largely positive and concentrated within a subset of genomic regions rather than being uniformly distributed genome-wide. A discordant signal at a locus on chromosome 18 in the pairwise analysis may partly contribute to the modest genome-wide correlation. In contrast, genome-wide genetic correlations between AD and MG subtypes were non-significant, likely reflecting limited statistical power or increased heterogeneity rather than an absence of shared genetic influences [35]. At the locus level, subtype-specific patterns were observed, with stronger and more widespread local correlations for AD–LOMG compared to AD–EOMG. AD–LOMG signals extended across both MHC and non-MHC loci, whereas AD–EOMG associations were more modest and largely confined to the MHC, suggesting a more restricted pattern of immune-mediated overlap for EOMG. These differences are consistent with known clinical and immunogenetic distinctions between EOMG and LOMG [49]. Several non-MHC loci in the AD–LOMG analyses showed local correlation signals, which should be interpreted as regional genetic concordance or discordance rather than evidence of specific causal genes or pathways. Overall, the results indicate a heterogeneous and locus-specific genetic architecture underlying AD–MG overlap, with greater breadth of involvement in LOMG than in EOMG. Taken together, these findings illustrate how predominantly concordant local effects, counterbalanced by a smaller number of discordant loci, may contribute to modest overall polygenic overlap while generating substantial regional heterogeneity. More broadly, the findings illustrate how local correlation approaches can uncover biologically meaningful signals that are potentially masked at the genome-wide level [35,50], revealing a complex and partly subtype-specific interplay between AD and MG that extends beyond classical immune loci. These findings may further support the interpretation that MG should not be considered a genetically uniform entity in the context of AD overlap.

Cross-trait meta-analysis refined this picture by identifying genome-wide significant variants that were not detected in the individual GWAS but emerged after meta-analysis. We identified additional putatively shared loci that were significant for either AD or MG and showed evidence of association with the other under a heterogeneity-aware meta-analytic framework. Independent SNPs were interpreted using a combination of BE *p*-values and trait-specific *m*-values, allowing distinction between loci supported by both traits and those primarily driven by one trait with secondary evidence in the other. A locus on chr16, represented by rs889555, previously associated with AD [51,52,53] emerged as putatively novel for MG. A second variant at this locus (rs59735493) also showed evidence of association with MG; however, these SNPs are in strong LD (r^2^ ≈ 0.94), indicating a single underlying regional signal rather than independent associations. The locus maps to the *BCKDK* gene involved in branched-chain amino acid metabolism, with reported relevance to neuronal and metabolic regulation [54,55]. Although MG associations at this locus were modest, they were accompanied by low or ambiguous *m*-values, and should be interpreted cautiously as suggestive of cross-trait overlap rather than evidence of a shared causal mechanism. Additional signals were observed at established AD loci [31], including regions on chrs 7 and 19, which also showed evidence of association with MG. Conversely, MG-associated MHC variants demonstrated strong associations with AD, consistent with immune-related loci contributing to the observed AD–MG overlap [20,56,57]. These findings support region-level convergence within a heterogeneous genetic architecture rather than definitive shared causal variants.

Colocalisation analyses indicated that most shared regions harbour distinct but closely linked causal variants, including within the MHC, pointing to locus-level rather than causal variant sharing. By contrast, the chromosome 16 locus at chr16: 29.03–31.38 Mb showed strong support for a shared causal variant, providing one of the clearest examples of sharing between the AD and MG. This region aligns with a significant positive local genetic correlation detected by LAVA at chr16: 29–31 Mb, providing cross-method support for shared causal variant at this locus. The predominance of PPA4 signals in our analysis, however, suggests that shared genomic regions between AD and MG generally reflect distinct causal variants operating within the same loci [36]. The finding underscores that regional overlap does not imply shared causal mechanisms and is consistent with a heterogeneous, locus-specific architecture.

Gene-based analyses provided complementary support by identifying convergent gene-level association signals across several loci, most prominently within the extended MHC region (including *HLA-DQB1*, *HLA-DRB1*, *HLA-DQA1*, and *HLA-DRA*). Outside the MHC, gene-level analyses implicated additional genes on chrs16 and 19, such as *ZNF668*, *CFAP119*, *POLR2E*, and *ABCA7*; several of these are known AD genes [53], but not previously linked to MG (to the best of our knowledge). Expression-based SMR analyses further strengthened these findings by identifying genes whose regulatory variation may influence both disorders, with fifteen genes showing shared evidence, primarily in the MHC and on chr16 (including *KAT8*, *PRSS36*, *YPEL3*, *PPP4C*, and *STX4*). Although these represent putatively shared genes, convergence at the gene level does not necessarily imply concordant variant-level effects, as aggregation can mask heterogeneity among individual SNPs. Local correlation analyses provide insights into these relationships: AD–MG showed largely positive effects, whereas LOMG and EOMG displayed more heterogeneous patterns across the MHC and non-MHC regions.

Synthesising these multilayered findings, we conclude that shared AD–MG overlap is modest but consistently supported and concentrated within a subset of genomic regions. While MHC loci account for the largest share of gene- and pathway-level enrichment, non-MHC contributions are evident, particularly in expression-based SMR analyses. Chr16 shows the strongest and most consistent evidence of cross-trait correlation, with colocalisation results consistent with a shared causal variant, whereas the MHC displays a mixture of putatively shared and occasionally opposing effects, particularly across MG subtypes. The combined evidence supports region-level biological convergence involving immune regulation, neuronal–immune interactions, and vesicle-trafficking pathways, rather than widespread genome-wide sharing or uniform single-variant pleiotropy. Overlapping biological pathways, including antigen presentation, T-cell activation, and interferon signalling, further support evidence of a shared immune framework. However, partitioned heritability analyses revealed that AD appears more strongly influenced by innate immune dysregulation, whereas MG reflects adaptive immune mechanisms. Our findings thus suggest that AD–MG overlap reflects a differential skew along shared immune pathways rather than a simple mechanistic convergence. Although immune enrichment may partly reflect ageing-related processes, our analyses focus on genetic variation and therefore primarily capture inherited susceptibility rather than immune activation acquired with age.

Bidirectional MR analyses provided additional insight, showing no evidence that genetic liability to AD increases risk of MG. In contrast, liability to MG yielded suggestive (but inconclusive) evidence of an increased risk of AD across multiple MR methods, although the estimated effect was cautiously interpreted. This signal remained directionally consistent when using an expanded set of MG instruments, with sensitivity analyses showing no evidence of horizontal pleiotropy or heterogeneity. While the estimate still warrants cautious interpretation, its consistency across methods broadly aligns with evidence linking autoimmune conditions to neurodegenerative risk. For example, previous MR analyses have reported causal associations between AD and certain autoimmune disorders, including multiple sclerosis [30], as well as between MG and other autoimmune diseases [58]. To the best of our knowledge, the present study represents the first MR-based assessment of causality between AD and MG. Current findings may suggest the possibility that immune-related genetic mechanisms contribute to downstream neurodegenerative vulnerability.

Finally, gene–drug interaction analyses highlighted potential translational relevance. Shared AD–MG genes showed predicted interactions with multiple approved or investigational agents, including anticoagulants (via *VKORC1*), immunomodulatory therapies targeting HLA class II genes, vesicle-trafficking-related agents interacting with syntaxin genes such as *STX4*, and Notch-pathway inhibitors linked to *NOTCH4*. These findings highlight immune- and synaptic-related pathways as potential targets for therapeutic investigation and suggest opportunities for further research informed by shared genetic architecture between AD and MG.

### Strengths and Limitations

This study has several methodological strengths. We used available large-scale GWAS datasets for AD and MG, thereby conducting a comprehensive evaluation of shared genetic architecture between the two disorders. We employed an integrative multi-layer analytical framework combining genome-wide correlation, locus-specific genetic correlation, SNP-level concordance, heterogeneity-aware cross-trait meta-analysis, colocalisation, gene-based aggregation, and expression-informed SMR analyses. This multi-resolution design enabled characterisation of AD–MG overlap across complementary genomic scales and allowed us to distinguish concordant and opposing genetic correlations, trait-specific signals, and locus-level convergence, patterns that cannot be resolved using any single analytical approach in isolation. Integration of tissue-specific functional annotations and expression-based causal inference further provided biological context for shared loci, facilitating interpretation of regulatory mechanisms operating in relevant immune and neural tissues. In addition, the application of stringent colocalisation and SMR criteria enabled the prioritisation of putative shared or distinct causal variants and genes, providing evidence beyond simple association-based overlap.

This study also has limitations. First, both GWAS datasets comprised individuals of European ancestry, which limits generalisability to other populations. Second, MG is a rare disease, and even the largest available GWAS includes only ~5700 cases, reducing power to detect smaller shared effects and limiting instrument strength for MR. As a result, our cross-trait estimates are likely conservative, and we recommend follow-up studies as more powerful MG data become available. Moreover, replication of the observed AD–MG overlap in future biobank-scale MG GWAS datasets will be important to confirm the robustness and generalisability of the findings, particularly for subtype-specific signals and non-MHC loci. Third, although we applied several MR approaches, limited MG instruments and residual horizontal pleiotropy cannot be ruled out. The suggestive MG→AD causal effect did not replicate in a smaller independent AD GWAS, likely reflecting reduced power; nonetheless, consistency across methods indicates that this signal merits cautious interest and future validation in larger datasets. Fourth, the MHC region contains complex LD with possible multiple causal variants, and single signal colocalisation models may oversimplify this architecture; thus, MHC interpretations should be treated with caution pending conditional fine mapping. Fifth, given recognised sex differences in both AD and MG, the relationship between these traits may be sex-specific; however, we were unable to assess this relationship in the present study owing to data limitations. Future studies using sex-stratified GWAS for both traits will be important to determine whether their shared genetic architecture differs by sex. Similarly, future studies comparing AD with other dementia subtypes in the context of MG would be valuable for clarifying disease specificity. Sixth, well-studied immune and neural cell types are more comprehensively represented in current databases; hence, tissue and pathway enrichment results should be interpreted in light of unequal annotation coverage across tissues. Seventh, we could not determine whether the observed overlap with AD was specific to MG or reflected broader immune-related genetic architecture because formal comparison with other autoimmune traits was beyond the scope of this study. Finally, gene–drug interaction findings are preliminary and require experimental validation, including assessment of target modulation in relevant cellular or model systems.

## 4. Materials and Methods

### 4.1. Data Source

We analysed a well-characterised publicly available AD GWAS comprising 71,880 cases and 383,378 controls, including both clinically diagnosed and AD-by-proxy cases [31]. The AD-by-proxy component was derived primarily from the UK Biobank, and it included individuals with a parental history of AD. A strong genetic correlation between clinically diagnosed AD and AD-by-proxy phenotypes (r ≈ 0.81) [22,31], support their combined use in genetic analyses. For replication, we used GWAS data restricted to clinically diagnosed AD (17,008 cases; 37,154 controls) [32]. For MG, we used a large GWAS dataset comprising 5708 cases and 432,028 controls, alongside subtype-specific summary statistics for EOMG: 1391 cases; 22,407 controls, and LOMG: 2404 cases; 64,103 controls [20]. All participants were of European ancestry. To assess regulatory mechanisms, we integrated whole-blood eQTL data from eQTLGen (n = 31,684), GTEx whole blood, brain-region-specific eQTL data from GTEx v8 across 12 brain regions, and BrainMeta, a harmonised meta-analysis of brain eQTLs across multiple studies [59,60,61]. This framework enabled evaluation of both peripheral immune and central nervous system regulatory architectures. All GWAS summary statistics, gene annotations, and eQTL data used in this study were in the GRCh37/hg19 genome build. Additional details on all data sources are provided in Supplementary Note S1.

### 4.2. Linkage Disequilibrium Score Regression Analysis

We estimated cross-trait genetic correlation between AD and MG, including MG subtypes (EOMG and LOMG), using bivariate LDSC (version 1.0.1) [33]. We used HapMap3 SNPs and LD scores from the 1000 Genomes European reference. Genetic covariance intercepts were constrained after confirming non-significant sample overlap. Multiple testing was corrected using the Bonferroni method. We performed sensitivity analyses excluding *APOE* and MHC regions. Supplementary Note S1 provides additional information on the methods.

### 4.3. Local Genetic Correlation Assessment

We performed local genetic correlation analyses using the LAVA framework (version 0.1.5) [35] to identify loci contributing disproportionately to AD–MG overlap, including MG subtypes: EOMG and LOMG [35,50,62]. The genome was partitioned into LD blocks using the 1000 Genomes European reference (MAF > 0.5%). Following allele harmonisation and univariate quality control, loci with sufficient signal were advanced to the bivariate testing. Analyses included a combined multi-trait model (AD, MG, EOMG, LOMG) and separate pairwise models to maximise SNP coverage [35,50,62]. For the pairwise LAVA analyses, the number of bivariate tests was determined by the number of loci that passed the univariate local heritability filter (*p* < 0.05) in both traits under comparison. This resulted in 35 loci for AD–MG, 24 loci for AD–EOMG, and 24 loci for AD–LOMG, and Bonferroni correction was applied accordingly using these denominators (Supplementary Note S1).

### 4.4. Assessing SNP Effect Concordance Between AD and MG

We assessed SNP effect concordance between AD and MG using SECA, testing whether independent SNPs associated with one trait (dataset 1) showed consistent effect directions in the other (dataset 2) [34]. Following quality control and allele harmonisation, LD clumping was applied to dataset 1 to derive independent SNPs, which were then stratified into 12 association *p*-value subsets and evaluated against dataset 2. Fisher’s exact tests, together with 1000 permutations, were used to assess the significance of effect concordance. Analyses were performed bidirectionally (AD → MG and MG → AD) to account for potential asymmetry in shared genetic effects [34]. Additional methodological details are provided in Supplementary Note S1.

### 4.5. Tissue- and Cell-Type-Specific Heritability Enrichment Analysis

We evaluated tissue- and cell-type-specific heritability enrichment for AD and MG using S-LDSC with SEG annotations, based on the framework of Finucane et al. [63]. This approach tests whether SNP heritability is enriched in SNPs mapped to genes specifically expressed in predefined expression-derived tissue or cell-type annotations [63,64]. Analyses incorporated baselineLD v2.2 annotations and LD scores from the 1000 Genomes European reference panel, covering a broad range of tissues and cell types with particular relevance to immune and neural systems. Nominal enrichment was defined as *p* < 0.05 (Supplementary Note S1).

### 4.6. Cross-Disorder GWAS Meta-Analysis and Characterisation of Genomic Loci

We conducted a heterogeneity-aware cross-disorder GWAS meta-analysis combining AD and MG summary statistics using METASOFT v2.0.1, applying the modified random-effects (RE2) model to identify loci consistent with shared effect [46,65]. In line with practice in previous studies [22,66,67], we aimed to identify variants not GWS in either of the individual GWAS (i.e., 5 × 10^−8^ < P_GWA-SNP_ < 0.001) but reached this status in meta-analysis (P_meta-analysis_ < 5 × 10^−8^). We also identify known loci showing cross-trait evidence. Binary effect and *m*-value frameworks were applied to assess trait-specific effects and account for heterogeneity [65] (Supplementary Note S1).

Post-meta-analysis, associated variants were annotated in FUMA [68] to identify independent SNPs and genomic loci. SNPs surpassing GWS (*p* < 5 × 10^−8^) in the meta-analysis but not in individual GWAS were LD-pruned (r^2^ < 0.6) to identify independent signals, and lead SNPs were defined at r^2^ < 0.1. Loci were assigned as ±250 kb around lead SNPs, with overlapping lead SNPs grouped into single loci. Identified SNPs/loci were cross-referenced with the GWAS Catalog to contextualise findings (Supplementary Note S1).

### 4.7. Assessing Causal Relationships Using Bidirectional MR

We applied bidirectional two-sample MR to investigate causal effects between AD and MG, adhering to STROBE-MR guidelines [37,38,69]. We performed analysis using the two-sample MR software (version 0.6.9). IVs were selected from GWAS summary statistics at GWS (*p* < 5 × 10^−8^), followed by LD clumping (r^2^ < 0.001, 10,000 kb window), and harmonisation to ensure alignment of effect alleles and reduce bias due to LD. To address weak instrument bias, we assessed instrument strength using F-statistics. Primary causal estimates were obtained using the IVW MR, complemented by weighted median and MR-Egger models. We conducted comprehensive sensitivity and further MR analyses [37,39,40,66,67,70,71,72]. These included Cochran’s Q statistic to evaluate heterogeneity in SNP effects, single-SNP MR analyses to examine the consistency of causal estimates across individual IVs, and leave-one-out analyses to determine whether any single IV disproportionately influenced the overall results. We applied the MR-Egger intercept test to assess deviations from the assumption of no directional pleiotropy, where a significant departure from zero would indicate a potential violation. We also used the MR-PRESSO method, which detects and removes outlier variants contributing to pleiotropic effects [40]. In addition, we performed bidirectional GSMR analyses with the HEIDI-outlier test [39]. IVs were selected using both genome-wide significance (*p* < 5 × 10^−8^) and suggestive (*p* < 1 × 10^−6^) thresholds to improve instrument strength and coverage (see Supplementary Note S1 for details).

### 4.8. Assessing Shared Loci of AD with MG in Colocalisation Analysis

To identify loci consistent with shared causal variants between AD and MG, we performed colocalisation analysis using GWAS-PW. This framework estimates four scenarios per genomic region: association with AD only (PPA1), MG only (PPA2), shared causal variant (PPA3), or independent variants for each trait (PPA4) [36]. Summary statistics were harmonised, merged by rsID and analysed within LD-defined regions from the 1000 Genomes European panel (see Supplementary Note S1).

### 4.9. Gene-Based Association and Independent Gene-Based Analyses

To assess whether AD and MG shared more associated genes than expected by chance, we performed a multi-step gene-level analysis integrating LD-aware gene-based association testing, independent gene estimation, and cross-trait overlap testing. We performed gene-based association analyses for AD and MG using fastBAT, mBAT, and mBAT-combo (v1.94.1) [41,73]. SNPs were assigned to genes using a ±50 kb window. These gene-based results were used in downstream analyses to identify putatively shared genes across traits, in line with a previous study [74] (Section 4.9.1). To account for LD among SNPs and neighbouring genes, we performed independent gene-based analyses using GEC [42], which estimates the effective number of independent genes. The output was subsequently used to assess gene-level overlap between AD and MG (Section 4.9.2).

#### 4.9.1. Identifying Putatively Shared Genes

To identify putatively shared genes, we integrated mBAT-combo gene-based association signals from AD and MG, retaining genes that met both nominal (P_gene_ ≤ 0.01) and multiple-testing-adjusted (FDR P_gene_ ≤ 0.05) significance thresholds in each trait. Gene-level evidence was then combined across AD and MG using Stouffer’s Z-score method [75], which evaluates whether moderate association signals occurring in both traits jointly support cross-trait association. Unlike Fisher’s method, which may be disproportionately influenced by a single extremely small *p*-value, Stouffer’s method averages standardised Z-scores and is therefore less sensitive to outlier signals. Gene-level evidence was combined with equal weighting assigned to both traits. Consequently, only genes showing evidence of association in both AD and MG contributed to statistically significant combined signals (Supplementary Note S1).

#### 4.9.2. Independent Gene-Based Test and Estimating Gene-Level Overlap

We quantified gene-level overlap between AD and MG by testing whether the observed number of shared associated genes exceeded that expected by chance, while accounting for LD-induced dependence among neighbouring genes [66,67,76,77,78,79]. Gene-based association analyses were first performed using the GATES method [47,48], which generates results suitable for independent gene-level testing. SNPs overlapping AD and MG were mapped to NCBI genes (build 37), and the most significant SNP per gene was retained as input for GEC analysis. Because neighbouring genes may be correlated through LD, we used GEC to estimate the effective number of approximately independent gene tests rather than assuming independence among all genes. GEC partitions markers into approximately independent LD blocks (r^2^ < 0.1), estimates the effective number of independent gene tests, and applies multiple-testing correction to control type I error [66,67,76,77,78,79]. The resulting effective gene counts were used to evaluate overlap between AD- and MG-associated genes. Gene sets were defined at three nominal significance thresholds (P_gene_ < 0.1, 0.05, and 0.01). Observed overlap was then compared with the overlap expected under the null hypothesis using a one-sided exact binomial test, with AD treated as the discovery set and MG as the target set (Supplementary Note S1).

### 4.10. Summary-Data-Based MR, Pathway, and Gene-Drug Analysis

We used SMR to integrate GWAS and eQTL data to identify potentially causal genes shared by AD and MG [80,81]. HEIDI testing was applied to distinguish pleiotropy from linkage, with significant associations defined by pHEIDI > 0.01 together with FDR-corrected SMR *p*-values. Analyses focused on blood-derived eQTLs from eQTLGen [59], GTEx whole blood, twelve GTEx v8 brain regions, and BrainMeta [60,61], capturing both peripheral immune and central nervous system regulatory mechanisms relevant to MG and AD [82,83]. Prioritised genes were subsequently examined using pathway enrichment analysis in g: Profiler (g:GOSt) [43,44], and potential therapeutic targets were assessed through the Drug–Gene Interaction Database (DGIdb) [45]. To increase robustness, only genes supported by at least two analytical approaches were retained for downstream biological interpretation. Further methodological details are provided in Supplementary Note S1.

## 5. Conclusions

This study provides, to our knowledge, the first comprehensive genetic investigation of the relationship between AD and MG, integrating genome-wide correlation, locus-specific analyses, gene-based and expression-informed approaches, pathway enrichment, MR, and gene–drug annotation. Across these complementary methods, we observed modest and nominal evidence of genome-wide polygenic overlap together with more consistent locus- and gene-level convergence, supporting limited but genuine shared genetic susceptibility. Locus-level analyses revealed predominantly positive local genetic correlations, but with heterogeneous effect patterns across MG subtypes. These findings suggest that modest global overlap is accompanied by region-specific genetic convergence concentrated within a subset of loci, including a chromosome 16 region and segments of the extended MHC, where evidence supports a mixture of shared, distinct, and occasionally opposing effects rather than uniform causal mechanisms.

Collectively, these results are consistent with partial convergence on immune-related genetic architecture, but in a heterogeneous and context-dependent manner rather than as a single shared biological pathway. Enrichment of immune-related genes and pathways likely reflects overlapping but non-specific immunogenetic contributions, while subtype differences in local genetic correlations point to distinct patterns of susceptibility between early- and late-onset MG. Notably, partitioned heritability analyses suggest that AD is more strongly influenced by innate immune dysregulation, whereas MG reflects adaptive immune mechanisms, indicating a differential skew along shared immune pathways. Bidirectional MR analyses provided no evidence for a causal effect of AD liability on MG and only weak, inconclusive indications in the reverse direction, suggesting that any shared genetic architecture does not translate into strong directional causality. Finally, the integration of genetic, transcriptomic, and pathway data highlights candidate loci, regulatory mechanisms, and biological pathways that may warrant further functional investigation. Overall, our findings support a model in which modest polygenic overlap coexists with heterogeneous, locus-specific genetic architecture linking AD and MG, with implications for understanding shared and distinct disease mechanisms.

## Figures and Tables

**Table 1:** Multi-trait local genetic correlation between Alzheimer’s disease and myasthenia gravis.

Significant (*p* < 3.42 × 10^−4,^ Bonferroni Corrected for 146 Analyses)										
Locus	Chr	Start	Stop	n.snps	phen1	phen2	rho	Rho (Lower)	Rho (Upper)	*p*
965	6	32,586,785	32,629,239	206	AD	MG	0.34	0.18	0.51	9.20 × 10^−5^
966	6	32,629,240	32,682,213	490	AD	LOMG	0.51	0.30	0.71	3.01 × 10^−5^
**Suggestively significant (3.42 × 10^−4^ < *p* < 0.05)**										
964	6	32,539,568	32,586,784	236	AD	MG	0.33	0.15	0.51	8.01 × 10^−4^
464	3	47,588,462	50,387,742	2442	AD	MG	0.75	0.28	1.00	3.33 × 10^−3^
966	6	32,629,240	32,682,213	490	AD	MG	0.24	0.02	0.47	3.56 × 10^−2^
952	6	27,261,036	28,666,364	2632	AD	MG	0.22	0.01	0.44	3.92 × 10^−2^
2135	16	53,393,883	54,866,095	2294	AD	LOMG	0.54	0.23	1.00	8.90 × 10^−4^
2255	18	20,009,697	21,622,716	2235	AD	LOMG	−0.28	−0.55	−0.04	2.51 × 10^−2^
959	6	31,250,557	31,320,268	984	AD	LOMG	0.55	0.06	1.00	3.94 × 10^−2^
956	6	30,070,718	30,715,006	2277	AD	LOMG	−0.48	−1.00	−0.03	4.50 × 10^−2^
962	6	32,208,902	32,454,577	1776	AD	LOMG	0.30	0.01	0.59	4.90 × 10^−2^
965	6	32,586,785	32,629,239	206	AD	EOMG	0.24	0.09	0.39	1.99 × 10^−3^
950	6	25,684,630	26,396,200	1714	AD	EOMG	0.29	0.06	0.52	1.30 × 10^−2^
964	6	32,539,568	32,586,784	236	AD	EOMG	0.21	0.03	0.39	2.51 × 10^−2^
956	6	30,070,718	30,715,006	2277	AD	EOMG	0.29	0.04	0.58	3.04 × 10^−2^

AD: Alzheimer’s disease, MG: Myasthenia gravis, EOMG: early onset myasthenia gravis, LOMG: Late onset myasthenia gravis, chr: chromosomes, n.snps: number of single nucleotide polymorphisms, phen: phenotype, rho (ρ) is the local genetic correlation between phen1 and phen2 at a locus (−1 to 1), with positive values indicating shared risk, negative values indicating opposite effects, and magnitude reflecting the strength of sharing, *p*: *p*-value.

**Table 2:** Pair-wise local genetic correlation of AD with MG and MG subtypes.

Locus	Chr	Start	Stop	n.snps	phen1	phen2	Rho	Rho Lower	Rho Upper	*p*
**AD-MG: Significant (*p* < 1.43 × 10^−3^** **, adjusting for 35 analyses)**										
2135	16	53,393,883	54,866,095	3486	AD	MG	0.54	0.27	1.00	1.27 × 10^−4^
965	6	32,586,785	32,629,239	360	AD	MG	0.19	0.07	0.31	1.58 × 10^−3^
**AD-MG: Suggestive (1.43 × 10^−3^ > *p* > 0.05)**										
954	6	29,529,756	29,833,843	2411	AD	MG	0.35	0.04	0.67	2.83 × 10^−2^
2255	18	20,009,697	21,622,716	2836	AD	MG	−0.23	−0.47	−0.02	3.55 × 10^−2^
**AD-LOMG: Significant (*p* < 2.08 × 10^−3^** **, adjusting for 24 analysis)**										
100	1	113,418,038	114,664,387	1514	AD	LOMG	0.90	0.75	1.00	8.24 × 10^−7^
964	6	32,539,568	32,586,784	311	AD	LOMG	0.79	0.66	0.89	1.04 × 10^−6^
965	6	32,586,785	32,629,239	285	AD	LOMG	0.50	0.34	0.63	5.01 × 10^−6^
1957	14	22,760,701	23,985,936	1822	AD	LOMG	0.81	0.64	0.97	1.11 × 10^−5^
959	6	31,250,557	31,320,268	1061	AD	LOMG	−0.62	−1.00	−0.31	4.26 × 10^−4^
1719	11	112,755,447	113,889,019	1873	AD	LOMG	0.89	0.65	1.00	6.39 × 10^−4^
**AD-LOMG: Suggestive (2.08 × 10^−3^ < *p* < 0.05)**										
958	6	31,106,494	31,250,556	1322	AD	LOMG	−0.46	−0.87	−0.15	4.36 × 10^−3^
2096	15	96,864,279	98,025,684	1280	AD	LOMG	0.62	0.29	0.86	5.53 × 10^−3^
2255	18	20,009,697	21,622,716	2267	AD	LOMG	0.63	0.28	0.85	5.58 × 10^−3^
955	6	29,833,844	30,070,717	1365	AD	LOMG	−0.60	−1.00	−0.12	1.71 × 10^−2^
960	6	31,320,269	31,427,209	1116	AD	LOMG	−0.33	−0.71	−0.04	2.64 × 10^−2^
**AD-EOMG: Significant (*p* < 2.08 × 10^−3^, adjusting for 24 analysis)**										
966	6	32,629,240	32,682,213	728	AD	EOMG	−0.23	−0.34	−0.13	1.26 × 10^−5^
969	6	33,194,976	33,864,262	1929	AD	EOMG	0.46	0.20	0.80	8.43 × 10^−4^
**AD-EOMG: Suggestive (2.08 × 10^−3^ < *p* < 0.05)**										
965	6	32,586,785	32,629,239	397	AD	EOMG	0.10	0.03	0.17	3.72 × 10^−3^
963	6	32,454,578	32,539,567	6	AD	EOMG	−0.49	−0.79	−0.17	5.73 × 10^−3^
950	6	25,684,630	26,396,200	1869	AD	EOMG	0.27	0.06	0.47	1.06 × 10^−2^
956	6	30,070,718	30,715,006	2444	AD	EOMG	0.29	0.05	0.54	1.69 × 10^−2^
1682	11	75,445,254	76,518,906	2284	AD	EOMG	−0.34	−0.81	−0.02	3.56 × 10^−2^

Chr: chromosomes, n.snps: number of single nucleotide polymorphisms, phen: phenotype, rho (ρ) is the local genetic correlation between phen1 and phen2 at a locus (−1 to 1), with positive values indicating shared risk, negative values indicating opposite effects, and magnitude reflecting the strength of sharing, *p*: *p*-value.

**Table 3:** Genome-wide significant independent SNPs and loci shared by AD and MG.

Independent SNPs	Unique ID	Genomic Loci	Lead SNPs	Individual GWAS *p*-Value		Meta-Analysis	Binary Effect *p*-Value	M-Value	
				AD	MG	*p*-Value (RE2)		AD	MG
Independent SNPs reaching genome-wide significance for AD and MG									
rs9268399	6:32340236:A:G	1	rs9268831	1.99 × 10^−7^	7.28 × 10^−5^	8.02 × 10^−9^	7.06 × 10^−8^	1.00	0.93
rs2076523	6:32370835:C:T			7.28 × 10^−8^	2.86 × 10^−4^	8.78 × 10^−9^	2.98 × 10^−8^	1.00	0.93
rs2395175	6:32405026:A:G			1.06 × 10^−7^	5.95 × 10^−4^	2.63 × 10^−8^	6.53 × 10^−8^	1.00	0.90
rs9268831	6:32427748:C:T			8.38 × 10^−8^	2.11 × 10^−6^	2.17 × 10^−10^	2.81 × 10^−8^	0.97	0.94
rs9270505	6:32559216:A:G			3.06 × 10^−6^	7.79 × 10^−7^	3.26 × 10^−9^	4.98 × 10^−7^	0.26	0.98
rs9270587	6:32561305:G:T			6.60 × 10^−8^	1.52 × 10^−5^	1.18 × 10^−9^	3.66 × 10^−8^	0.99	0.91
rs2858861	6:32580331:C:T			6.44 × 10^−8^	1.10 × 10^−3^	1.81 × 10^−8^	3.46 × 10^−8^	1.00	0.91
rs9271375	6:32587067:A:G			1.22 × 10^−7^	9.45 × 10^−6^	1.28 × 10^−9^	5.72 × 10^−8^	0.99	0.92
rs9271557	6:32590331:C:T			1.16 × 10^−7^	4.82 × 10^−5^	4.54 × 10^−9^	5.73 × 10^−8^	1.00	0.91
rs5002178	6:32611590:A:G			7.83 × 10^−7^	1.09 × 10^−5^	8.05 × 10^−9^	3.59 × 10^−7^	0.94	0.92
rs889555	16:31122571:C:T	2	rs889555	5.36 × 10^−8^	4.59 × 10^−4^	1.78 × 10^−8^	1.45 × 10^−7^	1.00	0.22
**Genome-wide significant AD-independent SNPs associated with MG**									
rs13201473	6:47489708:A:G	1	rs13201473	1.16 × 10^−8^	1.31 × 10^−2^	5.45 × 10^−9^	1.03 × 10^−8^	1.00	0.86
rs6979218	7:99893148:C:G	2	rs6979218	3.10 × 10^−12^	4.74 × 10^−2^	1.67 × 10^−12^	2.78 × 10^−12^	1.00	0.86
rs35251323	7:143095256:A:G	3	rs35251323	2.62 × 10^−10^	4.24 × 10^−3^	7.77 × 10^−11^	1.36 × 10^−10^	1.00	0.91
rs62472729	7:143116061:C:G		rs62472729	2.54 × 10^−8^	3.45 × 10^−2^	1.46 × 10^−8^	2.69 × 10^−8^	1.00	0.83
rs59735493	16:31133100:A:G	4	rs59735493	3.73 × 10^−8^	2.84 × 10^−4^	8.31 × 10^−9^	1.03 × 10^−7^	1.00	0.20
rs3752241	19:1053524:C:G	5	rs3752241	3.41 × 10^−10^	7.39 × 10^−3^	1.20 × 10^−10^	2.10 × 10^−10^	1.00	0.90
rs2965158	19:45195928:C:T	6	rs2965158	9.77 × 10^−9^	4.65 × 10^−2^	5.40 × 10^−9^	1.03 × 10^−8^	1.00	0.84
rs1871046	19:45351937:C:T		rs1871046	4.00 × 10^−22^	1.51 × 10^−2^	1.75 × 10^−22^	2.07 × 10^−22^	1.00	0.91
rs143668237	19:45486687:C:G		rs143668237	4.84 × 10^−24^	4.50 × 10^−2^	2.25 × 10^−24^	2.80 × 10^−24^	1.00	0.91
rs874744	19:45513417:C:T		rs874744	8.12 × 10^−27^	4.00 × 10^−2^	5.07 × 10^−27^	5.74 × 10^−27^	1.00	0.89
rs7251911	19:45582402:C:G		rs7251911	1.85 × 10^−10^	2.95 × 10^−2^	1.09 × 10^−10^	1.88 × 10^−10^	1.00	0.84
**Genome-wide significant MG-independent SNPs associated with AD**									
rs9271163	6:32577733:C:T	1	rs9271163	3.54 × 10^−8^	1.42 × 10^−9^	1.07 × 10^−13^	1.84 × 10^−10^	0.22	1.00
rs9271548	6:32590234:A:T		rs9271163	4.74 × 10^−8^	2.58 × 10^−9^	2.38 × 10^−13^	4.54 × 10^−10^	0.26	1.00

AD: Alzheimer’s disease, GWAS: genome-wide association studies, MG: myasthenia gravis, SNP: single-nucleotide polymorphism, RE2: modified random effect model of meta-analysis. For SNPs in each of these categories, we applied LD clumping using a threshold of r^2^ < 0.6 to define independent SNPs and further identified lead SNPs as those with r^2^ < 0.1 relative to others in the same region. Genomic loci were defined as regions within ±250 kb of each lead SNP, and overlapping regions were collapsed into a single locus; hence, there can be more than one lead SNP in a locus.

**Table 4:** Number of independent genes and gene-level overlap between AD and MG.

Traits	Discovery	Target	Overlapping Genes Between AD and MG	Proportion of Overlapping Genes Between AD and MG		Binomial Test *p*-Value
	AD	MG		Expected	Observed	
Total genes for AD and MG						
Raw number of genes	32,702	32,702	32,702			
Observed number of genes in GEC analysis	24,353	24,476	24,353			
Effective number of independent genes (GEC)	20,394	20,366	20,394			
Proportion of effective number of genes (GEC)	0.84	0.83	0.84			
**Genes with *p*-value ≤ 0.1**						
Raw number of genes	5037	5468	980	3023/20,366 = 0.148	577/2842 = 203	1.66 × 10^−15^
Observed number of genes in GEC analysis	3567	3833	704			
Effective number of independent genes (GEC)	2842	3023	577			
Proportion of effective number of genes (GEC)	0.80	0.79	0.82			
**Genes with *p*-value ≤ 0.05**						
Raw number of genes	3016	3389	465	1783/20,366 = 0.088	229/1640 = 140	4.33 × 10^−12^
Observed number of genes in GEC analysis	2106	2325	300			
Effective number of independent genes (GEC)	1640	1783	229			
Proportion of effective number of genes (GEC)	0.78	0.77	0.76			
**Genes with *p*-value ≤ 0.01**						
Raw number of genes	1122	1238	101	546/20,366 = 0.027	43/557 = 0.077	1.49 × 10^−9^
Observed number of genes in GEC analysis	764	785	66			
Effective number of independent genes (GEC)	557	546	43			
Proportion of effective number of genes (GEC)	0.73	0.70	0.65			

GEC: genetic type-1 error calculator, Raw number of genes: total number of genes obtained in the gene-based association analysis using the GATES method. Effective number of independent genes: the total number of independent genes obtained in the independent gene-based test using GEC.

**Table 5:** Genome-wide significant genes shared by AD and MG.

Gene	Chr Position (hg19) (Chr: Start–End)	AD			MG			Stouffer’s Method (Equal Weights)			
		Top SNP	Top SNP P	P_mBATcombo_	Top SNP	Top SNP P	P_mBATcombo_	Z-Score 1	Z-Score 2	Stouffer’s Z-Score	*p*
Genome-wide significant (sentinel) genes shared by AD and MG											
HLA-DQB1	6: 32,627,244–32,636,160	rs6931277	7.35 × 10^−11^	3.29 × 10^−11^	rs9271709	9.93 × 10^−17^	3.95 × 10^−25^	−6.53	−10.29	−11.89	6.45 × 10^−33^
BTNL2	6: 32,361,116–32,374,958	rs9469112	9.91 × 10^−11^	9.54 × 10^−12^	rs3117109	4.37 × 10^−19^	4.60 × 10^−22^	−6.71	−9.59	−11.52	4.95 × 10^−31^
TSBP1	6: 32,256,303–32,339,689	rs9268433	7.50 × 10^−10^	1.15 × 10^−10^	rs9268219	3.86 × 10^−19^	1.33 × 10^−21^	−6.34	−9.48	−11.18	2.48 × 10^−29^
HLA-DRA	6: 32,407,655–32,412,823	rs9469112	9.91 × 10^−11^	2.09 × 10^−12^	rs3129950	1.12 × 10^−18^	1.23 × 10^−19^	−6.93	−8.99	−11.26	1.05 × 10^−29^
HLA-DQA1	6: 32,595,956–32,614,839	rs6931277	7.35 × 10^−11^	1.81 × 10^−11^	rs9271709	9.93 × 10^−17^	3.96 × 10^−17^	−6.62	−8.33	−10.57	2.01 × 10^−26^
HLA-DRB1	6: 32,545,679–32,557,625	rs6931277	7.35 × 10^−11^	7.23 × 10^−11^	rs9271709	9.93 × 10^−17^	4.80 × 10^−17^	−6.41	−8.31	−10.41	1.13 × 10^−25^
HLA-DQA2	6: 32,709,168–32,714,975	rs9275477	4.09 × 10^−10^	1.90 × 10^−11^	rs9276625	1.29 × 10^−15^	2.85 × 10^−15^	−6.61	−7.81	−10.20	1.01 × 10^−24^
HLA-DQB2	6: 32,723,875- 32,731,309	rs3998159	6.45 × 10^−10^	4.02 × 10^−9^	rs9276625	1.29 × 10^−15^	2.83 × 10^−14^	−5.77	−7.52	−9.39	2.92 × 10^−21^
**AD genome-wide significant genes showing evidence of association with MG**											
CFAP119	16: 30,768,744–30,773,542	rs4889490	8.73 × 10^−6^	1.92 × 10^−6^	rs35695082	2.43 × 10^−5^	9.30 × 10^−4^	−4.62	−3.11	−5.47	2.29 × 10^−8^
ZNF668	16: 31,072,164–31,085,561	rs59735493	3.73 × 10^−8^	3.56 × 10^−7^	rs59735493	2.88 × 10^−4^	1.79 × 10^−3^	−4.96	−2.91	−5.57	1.31 × 10^−8^
ENSG00000 255439	16: 31,094,760–31,106,277	rs59735493	3.73 × 10^−8^	3.93 × 10^−7^	rs59735493	2.88 × 10^−4^	1.88 × 10^−3^	−4.94	−2.90	−5.54	1.5 × 10^−8^
ZNF646	16: 31,085,743–31,095,517	rs59735493	3.73 × 10^−8^	5.60 × 10^−7^	rs59735493	2.88 × 10^−4^	2.14 × 10^−3^	−4.87	−2.86	−5.46	2.35 × 10^−8^
VKORC1	16: 31,102,163–31,107,301	rs59735493	3.73 × 10^−8^	3.57 × 10^−7^	rs59735493	2.88 × 10^−4^	2.70 × 10^−3^	−4.96	−2.78	−5.47	2.21 × 10^−8^
PRSS53	16: 31,094,746–31,100,949	rs59735493	3.73 × 10^−8^	1.14 × 10^−6^	rs59735493	2.88 × 10^−4^	2.76 × 10^−3^	−4.73	−2.77	−5.31	5.63 × 10^−8^
ARHGAP45	19: 1,065,922–1,086,627	rs111278892	6.67 × 10^−11^	7.78 × 10^−13^	rs2868065	1.45 × 10^−4^	3.63 × 10^−3^	−7.07	−2.68	−6.90	2.65 × 10^−12^
POLR2E	19: 1,086,573–1,095,379	rs111278892	6.67 × 10^−11^	7.09 × 10^−15^	rs2868065	1.45 × 10^−4^	4.27 × 10^−3^	−7.69	−2.63	−7.30	1.43 × 10^−13^
ABCA7	19: 1,039,996–1,065,571	rs111278892	6.67 × 10^−11^	2.69 × 10^−12^	rs2868065	1.45 × 10^−4^	5.39 × 10^−3^	−6.89	−2.55	−6.68	1.21 × 10^−11^
GPX4	19: 1,103,993–1,106,790	rs4147929	4.43 × 10^−7^	2.45 × 10^−9^	rs2868065	1.45 × 10^−4^	8.00 × 10^−3^	−5.85	−2.41	−5.84	2.6 × 10^−9^
CNN2	19: 1,026,585–1,039,067	rs111278892	6.67 × 10^−11^	1.20 × 10^−12^	rs2868065	1.45 × 10^−4^	9.15 × 10^−3^	−7.01	−2.36	−6.62	1.74 × 10^−11^

AD: Alzheimer’s disease, MG: myasthenia gravis, chr: chromosome, *p*: *p*-value, SNP: single-nucleotide polymorphism, mBATcombo: The mBAT-combo is a gene-based analysis method that combines mBAT and fastBAT using a Cauchy combination approach, offering greater power than traditional sum-χ^2^ methods—especially for detecting genes with masking effects caused by complex linkage disequilibrium patterns.

## Data Availability

All data analysed in this study are fully described in the main manuscript and Supplementary Materials. GWAS summary statistics were obtained from publicly accessible repositories and international research consortia, as detailed in the data sources section. Supplementary Table S1 provides an overview of all datasets, including accession details or direct links for data access where appropriate.

## Supplementary Materials

The following supporting information can be downloaded at: https://www.mdpi.com/article/10.3390/ijms27114792/s1.
