# Supplementary material for: Genome-Wide and Locus-Level Analyses Reveal Modest, Heterogeneous Genetic Sharing Between Alzheimer’s Disease and Myasthenia Gravis
Source: Int J Mol Sci. 2026 May 26;27(11):4792. doi: 10.3390/ijms27114792 (PMC13256370; doi:10.3390/ijms27114792)
Supplement: Supplementary file 1 [file ijms-27-04792-s001.zip › Supplementary Note_S1_methods.pdf]

# Supplementary Note S1:

## Genome-wide and locus-level analyses reveal modest yet heterogeneous genetic sharing between Alzheimer's disease and myasthenia gravis


**Emmanuel O Adewuyi[1,2,3,4*], Asa Auta[5], Chinedu I. Ossai[6], Chidozie C. Anyaegbu[2,7], Thi Thu Huong Nguyen[1], Md Rezanur Rahman[8], Blossom C.M. Stephan[4], Gizachew A Tessema[1,2,9], Dale R Nyholt[10], Gavin Pereira[1,9,11]**

[1] Curtin School of Population Health, Faculty of Health Sciences, Curtin University, Perth, Western Australia, Australia (emmanuel.adewuyi@curtin.edu.au; thithuhuong.nguyen1@curtin.edu.au, gizachew.tessema@curtin.edu.au; and gavin.f.pereira@curtin.edu.au)

[2] Curtin Medical Research Institute, Faculty of Health Sciences, Curtin University, Perth, Western Australia, Australia (emmanuel.adewuyi@curtin.edu.au; chidozie.anyaegbu@curtin.edu.au, and gizachew.tessema@curtin.edu.au)

[3]Anchor University Centre for Global Health (AUCGH), Anchor University, Lagos, Nigeria (emmanuel.adewuyi@curtin.edu.au)

[4] Dementia Centre of Excellence, Curtin enAble Institute, Faculty of Health, Curtin University, Perth, Western Australia, Australia (emmanuel.adewuyi@curtin.edu.au, blossom.stephan@curtin.edu.au)

[5] Faculty of Health, Social Care and Medicine, Edge Hill University, Ormskirk, L39 4QP UK (asa.auta@edgehill.ac.uk)

[6] Independent Researcher – Melbourne, Victoria, Australia (ossaic@gmail.com)

[7] Perron Institute for Neurological and Translational Science, L1 Ralph and Patricia Sarich Neuroscience Research Institute, Nedlands, WA, Australia (chidozie.anyaegbu@curtin.edu.au)

[8] QIMR Berghofer Medical Research Institute, Herston, Brisbane, QLD, Australia (Rezanur.Rahman@qimrb.edu.au)

[9] Curtin enAble Institute, Curtin University, Perth, Western Australia, Australia (gizachew.tessema@curtin.edu.au; and gavin.f.pereira@curtin.edu.au)

[10] Centre for Genomics and Personalised Health, School of Biomedical Sciences, Faculty of Health, Queensland University of Technology, Brisbane, QLD, Australia (d.nyholt@qut.edu.au)

[11]Faculty of Medicine, Universitas Negeri Malang, Indonesia (gavin.f.pereira@curtin.edu.au)

*Corresponding author: emmanuel.adewuyi@curtin.edu.au

Supplementary Note 1: .......... 1
1.0 Overview of study design .......... 3
2.0 Data source .......... 4
3.0 Genome-wide genetic correlation assessment .......... 5
4.0 Local genetic correlation assessment .......... 6
5.0 Assessing SNP effect concordance .......... 6
6.0 Tissue-specific heritability enrichment analysis .......... 7
7.0 Cross-disorder GWAS meta-analysis .......... 7
8.0 Characterisation of independent SNPs and genomic loci .......... 8
9.0 Assessing causal relationships using Mendelian randomisation .......... 8
9.1 Instrumental variable selection for MR analysis .......... 9
9.2 MR statistical analysis .......... 9
9.3 Further MR and sensitivity analyses .......... 10
10.0 Assessing shared loci of AD with MG .......... 10
11.0 Gene-based association analysis .......... 11
11.1 Independent gene-based analysis .......... 11
11.2 Estimating gene-level overlap .......... 12
11.3 Identifying putative shared genes .......... 13
12.0 Summary data-based Mendelian randomisation .......... 13
13.0 Pathway enrichment analysis and gene-drug interactions .......... 14
14.0 References .......... 15

## 1.0 Overview of study design

We employ well-established and robust analytical approaches to investigate the genetic links between Alzheimer's disease (AD) and myasthenia gravis (MG) (Figure 1). First, we conducted a genome-wide cross-disease genetic correlation analysis using the linkage disequilibrium score regression (LDSC) method [1] to estimate the extent of shared polygenic architecture between AD, MG, Early-onset MG, and Late-onset MG. Beyond estimating the overall genome-wide genetic correlation with LDSC, we applied LAVA (Local Analysis of [co] Variant Association) [2] to identify specific genomic loci that contribute disproportionately to the genetic relationship between AD and MG, including its subtypes. Second, to further explore the consistency of genetic effects, we applied the single-nucleotide polymorphism (SNP) Effect Concordance Analysis (SECA) method [3], which assesses directional concordance and the likelihood of shared genetic influences at the SNP level across AD and MG. Third, we performed a cross-trait GWAS meta-analysis to identify genome-wide significant independent SNPs and genomic loci shared by AD and MG. This analysis was followed by a colocalisation analysis using the GWAS-PW (pairwise) method [4], which assessed 1,703 genomic regions to identify putatively shared loci with the same causal variants or loci harbouring distinct causal variants for each trait.

Fourth, we evaluated potential causal relationships between AD and MG using bidirectional Mendelian randomisation (MR) analysis [5-8]. We applied multiple MR models to strengthen causal inference, together with comprehensive sensitivity analyses and assessments of horizontal pleiotropy and heterogeneity to evaluate the validity of instrumental variable (IV) assumptions. Fifth, we performed gene-based association analyses to assess gene-level genetic overlap and identify genes putatively shared by AD and MG. We employed a robust framework incorporating three complementary models: fastBAT, mBAT, and mBAT-combo [9], which collectively enhance power to detect gene-level associations under varying patterns of LD. We also implemented the Genetic Type I Error Calculator (GEC)[10] for independent gene-based analysis. Furthermore, we applied Stouffer's method with equal weighting to combine gene-based p-values across both traits, thereby prioritising putatively shared genes supported by consistent evidence of association.

Sixth, to investigate the functional relevance of associated genes, we used the Summary data-based Mendelian Randomisation (SMR) approach to identify putative causal genes at shared loci. SMR integrates GWAS and expression quantitative trait loci (eQTL) data to assess whether genetically regulated gene expression is associated with disease risk [11, 12]. The HEIDI (heterogeneity in dependent instruments) test was employed to distinguish pleiotropy from linkage, supporting the prioritisation of candidate causal genes. Additionally, we conducted biological pathway enrichment analyses to identify key biological processes and molecular functions that may be implicated in both

disorders [13, 14]. Finally, we explored therapeutic relevance by mapping prioritised genes to known drug targets using gene–drug interaction databases. Our analyses were performed using well-powered data summarised in the methods section, with cohort-specific information provided in Supplementary Table 1.

## 2.0 Data source

We utilised large-scale GWAS summary statistics obtained from public repositories or international research consortia. For AD, we used one of the largest publicly available GWAS meta-analyses, comprising 71,880 AD cases and 383,378 controls [15]. This dataset integrates both clinically diagnosed AD cases and AD-by-proxy cases. The clinically diagnosed AD component consisted of multiple independent case–control cohorts, including approximately 24,000 clinically diagnosed AD cases and over 55,000 controls, totalling 79,145 participants, with case status determined using medical records or clinician-confirmed diagnoses [15]. Control individuals were defined as being free of AD or dementia at assessment. The AD-by-proxy component was derived primarily from the UK Biobank, and it included individuals with a parental history of AD. A strong genetic correlation between clinically diagnosed AD and AD-by-proxy phenotypes ($r \approx 0.81$) [15, 16], support their combined use in genetic analyses.

To test the potential replicability of our findings, we incorporated an additional GWAS dataset on clinically diagnosed AD cases [17]. This dataset was generated through meta-analyses of four major consortia: the Alzheimer's Disease Genetic Consortium, Cohorts for Heart and Aging Research in Genomic Epidemiology, the European Alzheimer's Disease Initiative, and the Genetic and Environmental Risk in Alzheimer's Disease Consortium [17]. The replication cohort (partial, as it is not completely independent) consisted of 17,008 AD cases and 37,154 controls [17].

For MG, we leveraged the largest available GWAS dataset [18], comprising 5,708 MG cases and 432,028 controls with a total sample size of 437,736. We also utilised age-of-onset-stratified data, available for only a subset of cohorts, comprising 1,391 cases and 22,407 controls for the early-onset MG GWAS, and 2,404 cases and 64,103 controls for the late-onset MG GWAS. The MG dataset was obtained through meta-analyses incorporating data from previously published and unpublished GWAS studies, along with summary statistics from large biobanks such as the UK Biobank, the Million Veteran Program, deCODE genetics, the Estonian Biobank, FinnGen, and BioVU. MG cases were identified using International Classification of Diseases (ICD) codes (ICD-10: G70.0; ICD-9: 358.0), ensuring diagnostic consistency across cohorts. Control participants were carefully screened to exclude common

autoimmune diseases, given their known genetic relationship with MG [19]. All included participants were of European ancestry. Further methodological details, including cohort characteristics, genotyping protocols, and quality control measures, can be found in the respective publications. A comprehensive summary of these GWAS datasets, including sample sizes and source references, is provided in Supplementary Table 1.

For eQTL data, we focused on both peripheral and central regulatory mechanisms relevant to MG and AD. To capture systemic immune processes implicated in MG pathogenesis, we used the eQTLGen consortium dataset, one of the largest blood eQTL resources available (~31,700 samples) [20]. For AD, where neuronal and glial mechanisms are central, we drew on the Genotype-Tissue Expression (GTEx) project (v8), which offers tissue-specific eQTL data across 13 brain regions [21]. From this resource, we selected twelve regions critical for cognition, memory, motor control, and neuroimmune regulation: hippocampus, frontal cortex (BA9), cerebellum, cerebellar hemisphere, hypothalamus, amygdala, substantia nigra, nucleus accumbens, caudate, putamen, and spinal cord (cervical c-1). To increase power and broaden coverage, we complemented GTEx with BrainMeta [22], a large-scale meta-analysis that harmonises eQTL findings across multiple brain studies. The combined use of eQTLGen, GTEx, and BrainMeta ensured that our analyses incorporated both peripheral immune and central nervous system regulatory architectures, providing a biologically informed framework for identifying putative shared causal genes between AD and MG.

## 3.0 Genome-wide genetic correlation assessment

We performed bivariate LDSC analyses [1] to estimate cross-trait genetic correlation (rg) between AD and MG. The LDSC approach quantifies trait heritability and genetic correlations by regressing GWAS test statistics on LD scores for each SNP, with the potential to distinguish genuine polygenic signals from confounding factors such as population stratification [1]. Pre-computed LD scores from the 1000 Genomes European reference panel were used for all LDSC analyses. Summary statistics for AD and MG were pre-processed to harmonise effect alleles and exclude SNPs with mismatched alleles or those with ambiguous strand orientation. We restricted our analysis to HapMap3 SNPs that intersect with the LD reference panel. We initially performed analyses with an unconstrained genetic covariance intercept to assess the proportion of potential sample overlap between AD and MG. The genetic covariance intercept was not significantly different from zero, indicating no evidence of significant sample overlap. Therefore, we proceeded with covariance intercept-constrained analyses. We applied the Bonferroni method for multiple testing correction. Additionally, we conducted sensitivity analyses excluding the *APOE* and MHC regions to examine their potential influence on AD-related results.

## 4.0 Local genetic correlation assessment

To identify genomic regions that may disproportionately contribute to the genetic overlap between AD and MG, we performed local genetic correlation analyses using the LAVA framework [2]. This approach complements genome-wide assessments by providing locus-specific insights [2]. Analyses included MG subtypes: EOMG and LOMG, and used the locus definitions from LAVA, which partition the genome into semi-independent LD blocks. The 1000 Genomes Project (v3) European panel (MAF > 0.5%) served as the LD reference. Following quality control, including harmonisation of effect alleles across all traits with the reference panel, LAVA first estimates local heritability for each trait at each locus. Only loci demonstrating adequate univariate signals, determined by their P-values, advanced to bivariate local genetic correlation analysis [2]. We used a relatively lenient filter ($P < 0.05$) to retain more loci for subsequent bivariate testing, consistent with previous studies [23, 24]. This approach enables a broader assessment of local genetic correlations without compromising statistical validity. Initially, we conducted a combined LAVA analysis including AD, MG, EOMG, and LOMG. To maximise the number of SNPs included, we also performed pairwise bivariate analyses for AD with MG, AD with EOMG, and AD with LOMG individually [23, 24]. LAVA only uses overlapping SNPs between traits, so analysing all traits simultaneously can reduce SNP coverage, justifying our use of separate pairwise analyses.

## 5.0 Assessing SNP effect concordance

We applied SECA [3] to assess the concordance of SNP effect directions between AD and MG. SECA provides insight into whether independent SNPs exhibit a consistent pattern of association across GWAS datasets. For this analysis, we designated the AD GWAS as dataset 1 and the MG GWAS as dataset 2. To ensure robust analysis, we performed rigorous quality control by removing non-rsID variants and duplicate SNPs. We then harmonised SNP effects by aligning alleles across both datasets, ensuring that effect sizes were measured relative to the same reference allele.

Next, we conducted LD clumping on dataset 1 to identify independent SNPs. This step was performed in two stages: (1) an LD threshold of $r^2 < 0.1$ within a 1 Mb window to capture independent signals, and (2) a broader window of 10 Mb to ensure long-range genetic effects were considered. The resulting independent SNPs were stratified into 12 subsets based on their association P-values ($P1 \leq 0.01$, 0.05, 0.1, 0.2, 0.3, 0.4, 0.5, 0.6, 0.7, 0.8, 0.9, 1.0). SECA then applied Fisher's exact test to assess SNP effect concordance across dataset 1 and dataset 2 [3]. This test involved evaluating whether SNPs in each of the 12 subsets from dataset 1 showed a consistent effect direction in dataset 2, yielding a $12 \times 12$ matrix (144 subset comparisons). A permutation-based correction (1,000 replicates) was applied to

adjust for multiple comparisons, ensuring the robustness of significance estimates. Analyses were performed bidirectionally (AD → MG and MG → AD) to account for potential asymmetry in genetic effects, whereby SNPs strongly associated with one trait may show weaker or non-significant effects in the other.

## 6.0 Tissue-specific heritability enrichment analysis

We used stratified LD score regression (sLDSC) cell-type-specific analysis (--h2-cts) to evaluate tissue- and cell-type-specific enrichment of SNP heritability for AD and MG. This framework integrates specifically expressed gene annotations to test whether SNP-heritability is enriched in genomic regions proximal to genes expressed in predefined tissues or cell types, thereby identifying biologically relevant systems that may contribute disproportionately to disease risk [25, 26]. Summary statistics were analysed using baselineLD v2.2 reference annotations together with tissue- and cell-type-specific gene sets, with LD scores and regression weights derived from the 1000 Genomes Project Phase 3 European reference panel. Analyses focused on immune- and neural-related tissues and cell-type annotations to clarify the tissue-specific genetic architecture of both disorders. Given the exploratory nature of this analysis, tissues and cell types with $P < 0.05$ were considered nominally enriched.

## 7.0 Cross-disorder GWAS meta-analysis

We performed a cross-disorder GWAS meta-analysis combining AD and MG summary statistics to identify SNPs and susceptibility loci that may be jointly implicated in both conditions. This analysis utilised two meta-analytic frameworks implemented in METASOFT: the fixed-effect (FE) model and the modified random-effects model (RE2) [27]. The FE model operates under the assumption that GWAS data for AD and MG are estimating the same underlying genetic effect, calculating P-values through inverse variance weighting. While efficient under homogeneity, this approach may be limited when heterogeneity exists in SNP effects. In contrast, the RE2 model accommodates variability in SNP effects across studies, providing increased power in scenarios where effect sizes differ between traits [27]. A total of 7,056,087 SNPs common to both AD and MG GWAS datasets were included in our meta-analysis, encompassing a combined sample size of 892,994 individuals.

In line with practice in previous studies [16, 28, 29], we focused on identifying variants that were not genome-wide significant (GWS) in either of the individual GWAS (i.e., $5 \times 10^{-8} < P_{GWA\text{-}SNP} < 0.001$) but reached the status following meta-analysis ($P_{meta\text{-}analysis} < 5 \times 10^{-8}$), as these may represent putatively

novel shared loci. Additionally, we examined loci previously associated with GWS in one trait that also showed evidence of association with the other. Significance thresholds were defined as $P_{meta\text{-}analysis} < 5 \times 10^{-8}$ for GWS and $P_{meta\text{-}analysis} < 1 \times 10^{-5}$ for suggestive evidence of association.

To further evaluate whether an association signal was likely present in each disorder, we applied the binary effect (BE) P-value and m-value frameworks [30]. The BE P-value is a frequentist measure that tests whether a SNP has an effect in at least one of the studies; a low value indicates strong evidence that the SNP is associated with one or both traits. Conversely, the m-value method uses cross-study posterior probability to assess the likelihood that a given SNP has an effect in individual datasets, particularly useful when heterogeneity is suspected. An m-value greater than 0.9 suggests strong evidence of an effect in a study, while values below 0.1 suggest no effect; intermediate values (0.1–0.9) reflect uncertainty [30].

## 8.0 Characterisation of independent SNPs and genomic loci

Following the cross-trait meta-analysis of AD and MG, we conducted a series of downstream analyses to annotate and interpret the associated genetic variants. As part of this process, we identified SNPs that were not GWS in either of the individual GWAS but surpassed the GWS threshold ($P < 5 \times 10^{-8}$) in the meta-analysis. From this set, we delineated independent significant SNPs using an LD threshold of $r^2 < 0.6$ and further defined lead SNPs as those with $r^2 < 0.1$ relative to other signals in the region. Genomic loci were defined as regions extending ±250 kb around each lead SNP. Lead SNPs located within the same 250 kb window were grouped into a single locus, allowing multiple lead variants to be assigned to one locus. Following the same process, we assessed whether loci previously reaching GWS in one trait showed evidence of association with the other. These analyses were performed using the FUMA (Functional Mapping and Annotation) web-based platform [31]. To further contextualise our findings, we cross-referenced the identified loci with the GWAS Catalog (https://www.ebi.ac.uk/gwas) to assess prior associations with AD and MG.

## 9.0 Assessing causal relationships using Mendelian randomisation

We applied a bidirectional two-sample MR framework to investigate whether genetic liability to AD causally influences MG risk, or vice versa. Multiple complementary MR methods were used to evaluate the robustness of potential causal relationships between the two traits. To strengthen the validity of the analysis, we selected appropriate IVs and conducted formal assessments of horizontal pleiotropy

and heterogeneity. Analyses were restricted to individuals of European ancestry and followed best-practice recommendations, including the STROBE-MR guidelines [5, 6, 32], with a completed checklist provided to ensure methodological rigour.

## *9.1 Instrumental variable selection for MR analysis*

We identified IVs from relevant GWAS summary statistics, selecting variants that reached genome-wide significance ($P < 5 \times 10^{-8}$) to ensure strong association with the exposure and an F-statistic > 10. This strategy minimised weak instrument bias and supported the relevance assumption of MR. The second assumption, namely that IVs are independent of confounders, is inherently more difficult to verify; however, we rigorously evaluated IV suitability and applied stringent linkage disequilibrium clumping ($r^2 < 0.001$, 10,000 kb window) to maximise instrument independence. To further improve data quality, we excluded variants with intermediate allele frequencies to minimise strand ambiguity, removed variants absent from the reference dataset, and harmonised exposure and outcome data by aligning alleles and preserving consistency in effect directions. Additional robustness checks were conducted as part of the main MR and sensitivity analyses.

## *9.2 MR statistical analysis*

In this study, we used the inverse-variance weighted (IVW) method as the primary MR approach. This method combines Wald ratios from individual genetic instruments into a single variance-weighted estimate, with weights determined by the inverse variance of each ratio. The IVW framework assumes the absence of horizontal pleiotropy and provides consistent estimates when this assumption is reasonably met. To accommodate potential heterogeneity in causal estimates across variants, we applied the multiplicative random-effects IVW model. To complement IVW estimates, we also implemented additional MR methods, including the weighted median estimator, which remains valid when up to 50% of the total instrument weight derives from invalid IVs, and MR-Egger regression, which allows for directional pleiotropy while providing pleiotropy-adjusted causal estimates.

We also implemented weighted mode and simple mode as additional methods. Results with $P < 0.05$ were considered nominally significant, whereas significance after multiple-testing correction was defined as $P < 0.025$ (Bonferroni correction for two directional tests). All SNP effect estimates were modelled on the log-odds scale for the binary traits AD and MG, with harmonised beta coefficients and standard errors used to derive Wald ratios and corresponding variance-weighted causal estimates. Data processing and analyses were conducted in the Unix environment using R and the TwoSampleMR package (version 0.5.6) implemented in R version 4.2.1.

### *9.3 Further MR and sensitivity analyses*

To ensure the reliability of our MR analyses and align with best practices in the field [5, 28, 29, 33-35], we conducted a comprehensive series of sensitivity tests. These included Cochran's Q statistic to evaluate heterogeneity in SNP effects, single-SNP MR analyses to examine the consistency of causal estimates across individual IVs, and leave-one-out analyses to determine whether any single IV disproportionately influenced the overall result, thereby helping to identify potential outliers or pleiotropic variants. We also examined funnel plot symmetry to detect possible bias. The MR-Egger intercept test was applied to assess deviations from the assumption of no directional pleiotropy, where a significant departure from zero would indicate a potential violation. To further account for pleiotropy, we used the MR Pleiotropy RESidual Sum and Outlier (MR-PRESSO) method, which detects and removes outlier variants contributing to pleiotropic effects [8]. In addition, we performed bidirectional Generalised Summary-data-based Mendelian Randomisation (GSMR) analyses [7]. IVs were selected using both genome-wide significance ($P < 5 \times 10^{-8}$) and suggestive ($P < 1 \times 10^{-6}$) thresholds to improve instrument strength and coverage. GSMR extends conventional MR by accounting for residual LD between SNPs and implementing the HEIDI-outlier test to identify and remove pleiotropic variants [7], thereby strengthening the robustness of causal inference by reducing bias from invalid instruments.

## 10.0 Assessing shared loci of AD with MG

To investigate potential shared genetic loci between AD and MG, we conducted a colocalisation analysis using the GWAS-PW method [4]. GWAS-PW systematically scans the genome to identify regions likely to harbour either a shared causal variant or distinct causal variants for the two traits [4]. This method estimates the probability of four possible scenarios for a given genomic region: (1) a variant is associated only with AD (PPA1), (2) a variant is associated only with MG (PPA2), (3) a variant is shared by AD and MG (PPA3), or (4) the region contains independent variants associated with each trait (PPA4) [4].

For this analysis, we first harmonised AD and MG GWAS summary statistics to ensure consistent allelic orientation. The datasets were merged using rsID and allele information, aligning effect and non-effect alleles across both traits. Standardised Z-scores and variances for each SNP were then used as input for the GWAS-PW model. Analyses were performed across predefined, approximately independent genomic regions based on LD patterns from the 1000 Genomes Project European reference panel. In addition, we confirmed that there was no substantial sample overlap between the AD and MG datasets, minimising potential bias arising from shared individuals across GWAS cohorts. Our primary

parameters of interest were PPA3 and PPA4. Regions with PPA3 > 0.9 were considered to show strong evidence of a shared causal variant between AD and MG, whereas PPA3 > 0.5 indicated moderate-to-high support for shared regional association. Similarly, regions with PPA4 > 0.9 were interpreted as harbouring distinct causal variants for each trait within the same genomic region, while PPA4 > 0.5 indicated moderate-to-high support for this pattern.

## 11.0 Gene-based association analysis

To extend our investigation from individual SNPs to the gene level, we performed gene-based association analyses using fastBAT, mBAT, and mBAT-combo [9, 36]. Gene-based analysis aggregates SNP effects within a gene to assess its overall contribution to a trait or disease. Rather than testing each SNP individually, gene-based methods integrate SNP-level data to enhance statistical power and biological interpretability. The mBAT-combo gene analysis method integrates mBAT and fastBAT statistics using a Cauchy combination approach, offering advantages over traditional sum-$\chi^2$ methods, particularly in detecting genes with masking effects, where LD patterns can obscure associations [9]. This method has demonstrated superior power in both simulations and real-world datasets, identifying more gene-trait associations than existing approaches [9]. Therefore, we prioritised its use in this study, especially for identifying shared genes and for downstream analysis of combining P-values. We performed analyses separately for AD and MG, using overlapping SNPs from their respective GWAS, following the same approach in a previous study [37]. SNPs were assigned to genes using a window size of 50 kilobases. The outputs of our gene-based analyses were used to identify putatively shared genes.

### *11.1 Independent gene-based analysis*

Gene-based analyses can provide valuable insights into the biology of complex traits [10]. However, LD among the most significant SNPs assigned to each gene may influence gene-based association results, as non-independence across adjacent genes can inflate association signals. To address this limitation and better estimate gene-level genetic overlap, we performed independent gene-based analyses using the GEC software [10], as applied in prior studies [28, 29, 38-41]. The GEC analysis serves two main purposes: mitigating correlations among neighbouring gene-based associations and generating unbiased data for quantifying gene-level overlap between AD and MG.

First, we conducted gene-based association analyses using the GATES method [42], implemented within the FAST framework [43], which is particularly suited for independent gene-level testing. Specifically, the minSNP-based test was applied, selecting the SNP with the smallest p-value assigned

to a gene and adjusting it for the effective number of independent SNPs, thereby accounting for LD. GATES estimates this effective number via an eigenvalue decomposition of the SNP × SNP LD correlation matrix [42]. We used the 1000 Genomes Project CEU as reference data (May 2012 release). Simulation studies have shown that GATES effectively controls type 1 error regardless of gene size or LD structure and does not require permutation to validate significance [42]. For this analysis, SNPs shared by AD and MG were mapped to 32,702 NCBI genes (build 37). The GATES output provided the most significantly associated SNP for each gene along with its adjusted p-value, forming the basis for downstream analyses for independent gene analysis and, subsequently, gene-level overlap.

Each gene's best-associated SNP, defined as the variant with the lowest GWAS P-value, was input into the GEC software for analysis. GEC accounts for LD among these SNPs by partitioning them into independent LD blocks ($r^2 < 0.1$) and performing an eigenvalue decomposition of the correlation matrix within each block. This procedure estimates the effective number of independent gene-based tests and applies multiple-testing correction to control type 1 error. Effective gene counts were estimated separately for AD and MG, allowing significance thresholds that reflect underlying LD structure. This approach enhances the robustness and interpretability of gene-based findings, as demonstrated in previous studies [28, 29, 38-41].

### *11.2 Estimating gene-level overlap*

We evaluated whether genes shared between AD and MG were observed more frequently than expected under a null model of random overlap, using three gene-based significance thresholds ($P_{gene} < 0.1$, 0.05, and 0.01). This analysis was based on results from the independent gene-based tests using the GEC, following the example in practice in previous studies [34, 39, 41, 44]. To account for LD among nearby genes, we first derived the effective number of independent genes that overlapped between AD and MG at each threshold [34, 39, 41]. AD was designated as the discovery phenotype, while MG served as the target phenotype. For each threshold, we quantified both the observed and expected proportions of overlapping genes across the two traits. Statistical significance of enrichment was assessed using a one-sided exact binomial test comparing observed and expected proportions across thresholds, testing whether overlap exceeded chance expectation. The expected proportion was defined as the fraction of genes meeting the threshold in the target set relative to the total effective number of independent genes in that set. In contrast, the observed proportion was calculated as the effective number of genes significant in both traits divided by the effective number of significant genes in the discovery set.

### *11.3 Identifying putative shared genes*

To enhance statistical power and identify putatively shared genes between AD and MG, we applied a gene-based p-value aggregation approach. Analyses were restricted to genes passing Benjamini–Hochberg false discovery rate (FDR) correction in the mBAT-combo gene-based results. Specifically, genes were included for cross-trait integration if they showed $P_{gene} \leq 0.01$ and BH-adjusted $P_{gene} \leq 0.05$ in both AD and MG. We combined gene-level p-values using Stouffer's Z-score method, which integrates association evidence by averaging Z-scores rather than combining log-transformed p-values, thereby reducing sensitivity to extreme outliers and prioritising concordant signals across traits [45]. Equal weights were assigned to AD and MG to ensure balanced contribution from both datasets. Consequently, the resulting combined statistics primarily reflect genes with consistent evidence of association across the two traits, improving prioritisation of shared genetic signals.

## 12.0 Summary data-based Mendelian randomisation

We conducted expression-based SMR analyses using GWAS summary statistics for AD and MG to prioritise putative shared genes whose expression is consistent with a single shared causal-variant model underlying risk for both disorders. SMR integrates GWAS summary statistics with expression quantitative trait locus (eQTL) data to test whether genetically predicted gene expression is associated with disease through shared genetic variation rather than linkage [11]. In SMR, cis-eQTL variants are used as IVs to estimate the association between gene expression and disease traits using summary-level GWAS and eQTL data, and analyses were performed using the SMR software package [11, 12]. For each gene, the most significant cis-eQTL variant within ±1 Mb of the transcription start site was selected as the IV. SMR analyses were conducted separately for AD and MG. The SMR effect estimate was calculated as the ratio of the SNP–phenotype association to the SNP–expression association. To distinguish putative pleiotropy from linkage, we applied the HEIDI test, which evaluates whether the pattern of association across nearby SNPs in LD is consistent with a single shared causal variant influencing both gene expression and disease risk. Gene–trait associations showing evidence of heterogeneity (pHEIDI < 0.01) were excluded as likely reflecting linkage rather than pleiotropy. Associations were considered significant if they survived FDR correction and satisfied pHEIDI > 0.01.

We focused on blood and brain tissues to capture regulatory mechanisms relevant to immune and neurobiological processes underlying AD–MG overlap. Blood provides a window into systemic immune regulation central to MG pathogenesis, and we therefore used eQTL summary statistics from eQTLGen [20], one of the largest available meta-analyses of blood eQTLs, alongside whole blood data from GTEx

v8. For AD, brain tissue is particularly relevant, as regulatory variation in neuronal and glial cell types may influence disease risk. We therefore used eQTL data from GTEx v8 brain tissues, including the hippocampus, frontal cortex, cerebellum, cerebellar hemisphere, hypothalamus, amygdala, substantia nigra, nucleus accumbens, caudate, putamen, and spinal cord [21, 22]. To improve power to detect regulatory variants that may be missed in individual brain regions, we complemented GTEx analyses with BrainMeta [22], which aggregates and harmonises brain eQTL results across multiple independent studies. Including both blood and brain eQTL resources enabled assessment of peripheral immune and central nervous system regulatory mechanisms contributing to shared AD–MG genetic architecture. Genes were prioritised as shared if they showed significant SMR associations with both AD and MG within the same eQTL dataset and tissue context, consistent with a common regulatory mechanism.

## 13.0 Pathway enrichment analysis and gene-drug interactions

To interpret the biological relevance of genes shared between AD and MG and to explore potential underlying mechanisms, we performed pathway enrichment analyses using the g: GOst function implemented in the g: Profiler platform [13]. Analyses focused on a curated set of genes prioritised as associated with both conditions and supported by at least two independent analytical approaches within our study. The g: GOst tool identifies enriched biological processes and functional categories by testing for over-representation of user-provided gene sets against curated functional databases, with appropriate correction for multiple testing [13]. Analyses followed established guidelines, with minimum and maximum term sizes set to 5 and 350 genes, respectively, to refine the functional categories considered [14]. Queries were restricted to human genes, and default advanced g: Profiler settings were retained. Statistical significance was assessed using the recommended g: SCS multiple-testing correction method, with pathways considered significant at an adjusted P-value < 0.05 [13, 14]. To explore potential therapeutic implications, we examined gene–drug interactions using the Drug–Gene Interaction Database (DGIdb; version 5.0.9, accessed July 10, 2025) [46]. This analysis focused on genes supported by at least two analytical approaches in our study and aimed to identify known or potential drug targets based on curated gene–drug interaction evidence.

## 14.0 References


1. Bulik-Sullivan, B.K., et al., *LD Score regression distinguishes confounding from polygenicity in genome-wide association studies.* Nat Genet, 2015. **47**(3): p. 291-5.

2. Werme, J., et al., *An integrated framework for local genetic correlation analysis.* Nature Genetics, 2022. **54**(3): p. 274-282.

3. Nyholt, D.R., *SECA: SNP effect concordance analysis using genome-wide association summary results.* Bioinformatics, 2014. **30**(14): p. 2086-2088.

4. Pickrell, J.K., et al., *Detection and interpretation of shared genetic influences on 42 human traits.* Nat Genet, 2016. **48**(7): p. 709-17.

5. Davies, N.M., M.V. Holmes, and G. Davey Smith, *Reading Mendelian randomisation studies: a guide, glossary, and checklist for clinicians.* BMJ, 2018. **362**: p. k601.

6. Richmond, R.C. and G. Davey Smith, *Mendelian Randomization: Concepts and Scope.* Cold Spring Harb Perspect Med, 2022. **12**(1).

7. Zhu, Z., et al., *Causal associations between risk factors and common diseases inferred from GWAS summary data.* Nature Communications, 2018. **9**(1): p. 224.

8. Verbanck, M., et al., *Detection of widespread horizontal pleiotropy in causal relationships inferred from Mendelian randomization between complex traits and diseases.* Nature genetics, 2018. **50**(5): p. 693-698.

9. Li, A., et al., *mBAT-combo: A more powerful test to detect gene-trait associations from GWAS data.* The American Journal of Human Genetics, 2023. **110**(1): p. 30-43.

10. Li, M.-X., et al., *Evaluating the effective numbers of independent tests and significant p-value thresholds in commercial genotyping arrays and public imputation reference datasets.* Human Genetics, 2012. **131**(5): p. 747-756.

11. Zhu, Z., et al., *Integration of summary data from GWAS and eQTL studies predicts complex trait gene targets.* Nature genetics, 2016. **48**(5): p. 481-487.

12. Guo, Y., et al., *SMR-Portal: an online platform for integrative analysis of GWAS and xQTL data to identify complex trait genes.* Nature Methods, 2025. **22**(2): p. 220-222.

13. Raudvere, U., et al., *g:Profiler: a web server for functional enrichment analysis and conversions of gene lists (2019 update).* Nucleic Acids Research, 2019. **47**(W1): p. W191-W198.

14. Reimand, J., et al., *Pathway enrichment analysis and visualization of omics data using g:Profiler, GSEA, Cytoscape and EnrichmentMap.* Nature Protocols, 2019. **14**(2): p. 482-517.

15. Jansen, I.E., et al., *Genome-wide meta-analysis identifies new loci and functional pathways influencing Alzheimer's disease risk.* Nature genetics, 2019. **51**(3): p. 404-413.

16. Adewuyi, E.O., et al., *A large-scale genome-wide cross-trait analysis reveals shared genetic architecture between Alzheimer's disease and gastrointestinal tract disorders.* Communications biology, 2022. **5**(1): p. 691.

17. Lambert, J.C., et al., *Meta-analysis of 74,046 individuals identifies 11 new susceptibility loci for Alzheimer's disease.* Nat Genet, 2013. **45**(12): p. 1452-8.

18. Braun, A., et al., *Genome-wide meta-analysis of myasthenia gravis uncovers new loci and provides insights into polygenic prediction.* Nature Communications, 2024. **15**(1): p. 9839.

19. Li, K., Y. Ouyang, and H. Yang, *Myasthenia gravis and five autoimmune diseases: a bidirectional Mendelian randomization study.* Neurological Sciences, 2024. **45**(4): p. 1699-1706.

20. Võsa, U., et al., *Large-scale cis-and trans-eQTL analyses identify thousands of genetic loci and polygenic scores that regulate blood gene expression.* Nature genetics, 2021. **53**(9): p. 1300-1310.

21. Consortium, G., et al., *The Genotype-Tissue Expression (GTEx) pilot analysis: multitissue gene regulation in humans.* Science, 2015. **348**(6235): p. 648-660.

22. Qi, T., et al., *Genetic control of RNA splicing and its distinct role in complex trait variation.* Nature Genetics, 2022. **54**(9): p. 1355-1363.

23. Adewuyi, E.O., et al., *Genome-wide cross-disease analyses highlight causality and shared biological pathways of type 2 diabetes with gastrointestinal disorders.* Communications Biology, 2024. **7**(1): p. 643.

24. Kirby, A., et al., *Investigating Genetic Overlap between Alzheimer's Disease, Lipids, and Coronary Artery Disease: A Large-Scale Genome-Wide Cross Trait Analysis.* International Journal of Molecular Sciences, 2024. **25**(16): p. 8814.

25. Finucane, H.K., et al., *Heritability enrichment of specifically expressed genes identifies disease-relevant tissues and cell types.* Nature genetics, 2018. **50**(4): p. 621-629.

26. Cuellar-Partida, G., et al., *Complex-Traits Genetics Virtual Lab: A community-driven web platform for post-GWAS analyses.* BioRxiv, 2019: p. 518027.

27. Han, B. and E. Eskin, *Random-effects model aimed at discovering associations in meta-analysis of genome-wide association studies.* The American Journal of Human Genetics, 2011. **88**(5): p. 586-598.

28. Adewuyi, E.O., D. Mehta, and D. Nyholt, *Genetic overlap analysis of endometriosis and asthma identifies shared loci implicating sex hormones and thyroid signalling pathways.* Human Reproduction, 2022. **37**(2): p. 366-383.

29. Islam, M.R., D.R. Nyholt, and C. The International Headache Genetics, *Cross-trait analyses identify shared genetics between migraine, headache, and glycemic traits, and a causal relationship with fasting proinsulin.* Human Genetics, 2023.

30. Han, B. and E. Eskin, *Interpreting meta-analyses of genome-wide association studies.* PLoS genetics, 2012. **8**(3): p. e1002555.

31. Watanabe, K., et al., *Functional mapping and annotation of genetic associations with FUMA.* Nature communications, 2017. **8**(1): p. 1826.

32. Skrivankova, V.W., et al., *Strengthening the reporting of observational studies in epidemiology using Mendelian randomization: the STROBE-MR statement.* Jama, 2021. **326**(16): p. 1614-1621.

33. Akosile, W. and E. Adewuyi, *Genetic correlation and causality assessment between post-traumatic stress disorder and coronary artery disease-related traits.* Gene, 2022. **842**: p. 146802.

34. Tasnim, S., et al., *Shared genetics and causal relationships between migraine and thyroid function traits.* Cephalalgia, 2023. **43**(2): p. 03331024221139253.

35. Adewuyi, E.O., et al., *Relationship of Cognition and Alzheimer’s Disease with Gastrointestinal Tract Disorders: A Large-Scale Genetic Overlap and Mendelian Randomisation Analysis.* International Journal of Molecular Sciences, 2022. **23**(24): p. 16199.

36. Bakshi, A., et al., *Fast set-based association analysis using summary data from GWAS identifies novel gene loci for human complex traits.* Scientific Reports, 2016. **6**(1): p. 32894.

37. Adewuyi, E.O. and S.M. Laws, *Genomic Characterisation of the Relationship and Causal Links Between Vascular Calcification, Alzheimer’s Disease, and Cognitive Traits.* Biomedicines, 2025. **13**(3): p. 618.

38. Adewuyi, E.O., et al., *Genetic analysis of endometriosis and depression identifies shared loci and implicates causal links with gastric mucosa abnormality.* Human Genetics, 2021. **140**(3): p. 529–552.

39. Adewuyi, E.O., et al., *Shared Molecular Genetic Mechanisms Underlie Endometriosis and Migraine Comorbidity.* Genes, 2020. **11**(3): p. 268.

40. Yang, Y., et al., *Molecular genetic overlap between migraine and major depressive disorder.* European Journal of Human Genetics, 2018. **26**(8): p. 1202-1216.

41. Zhao, H., et al., *Gene-based pleiotropy across migraine with aura and migraine without aura patient groups.* Cephalalgia, 2016. **36**(7): p. 648-57.

42. Li, M.-X., et al., *GATES: a rapid and powerful gene-based association test using extended Simes procedure.* The American Journal of Human Genetics, 2011. **88**(3): p. 283-293.

43. Chanda, P., et al., *Fast association tests for genes with FAST.* PloS one, 2013. **8**(7): p. e68585.
44. Adewuyi, E.O., et al., *Genetic analysis of endometriosis and depression identifies shared loci and implicates causal links with gastric mucosa abnormality.* Human genetics, 2021. **140**: p. 529-552.

45. Whitlock, M.C., *Combining probability from independent tests: the weighted Z-method is superior to Fisher's approach.* Journal of evolutionary biology, 2005. **18**(5): p. 1368-1373.

46. Cannon, M., et al., *DGIdb 5.0: rebuilding the drug–gene interaction database for precision medicine and drug discovery platforms.* Nucleic Acids Research, 2023. **52**(D1): p. D1227-D1235.
