# Supplementary material for: Genome-Wide and Locus-Level Analyses Reveal Modest, Heterogeneous Genetic Sharing Between Alzheimer’s Disease and Myasthenia Gravis
Source: Int J Mol Sci. 2026 May 26;27(11):4792. doi: 10.3390/ijms27114792 (PMC13256370; doi:10.3390/ijms27114792)
Supplement: Supplementary file 1 [file ijms-27-04792-s001.zip › Supplementary_figures.pdf]

Supplementary Figures

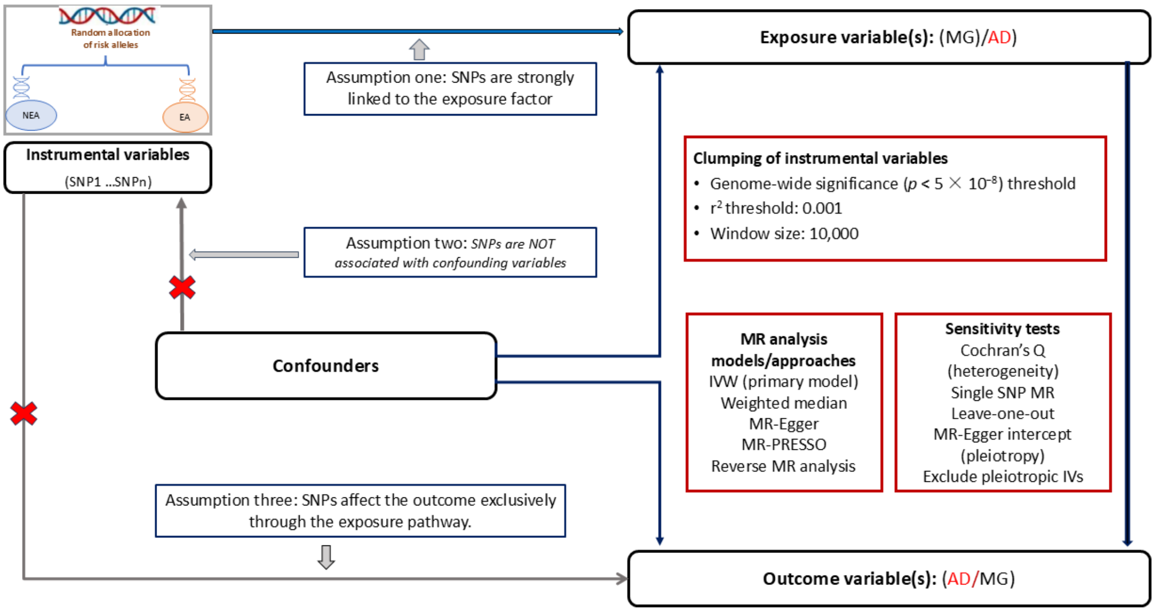

Supplementary Figure S1: MR framework and overview of assumptions. AD: Alzheimer's disease, EA: effect allele, IVW: inverse variance weighted, MG: Myasthenia gravis, MR: Mendelian randomisation, MR-PRESSO: Mendelian randomisation pleiotropy residual sum and outlier, NEA: non-effect allele, SNP: single nucleotide polymorphism. The figure outlines the MR analysis method, focusing on its use and key assumptions in exploring potential causal links between exposure and outcome variables. Additionally, the figure illustrates the clumping parameters employed to ensure the independence and relevance of the genetic instruments. The analysis was conducted in two phases: first, using MG as the exposure and AD as the outcome, and then reversing the roles, with AD as the exposure and MG as the outcome.

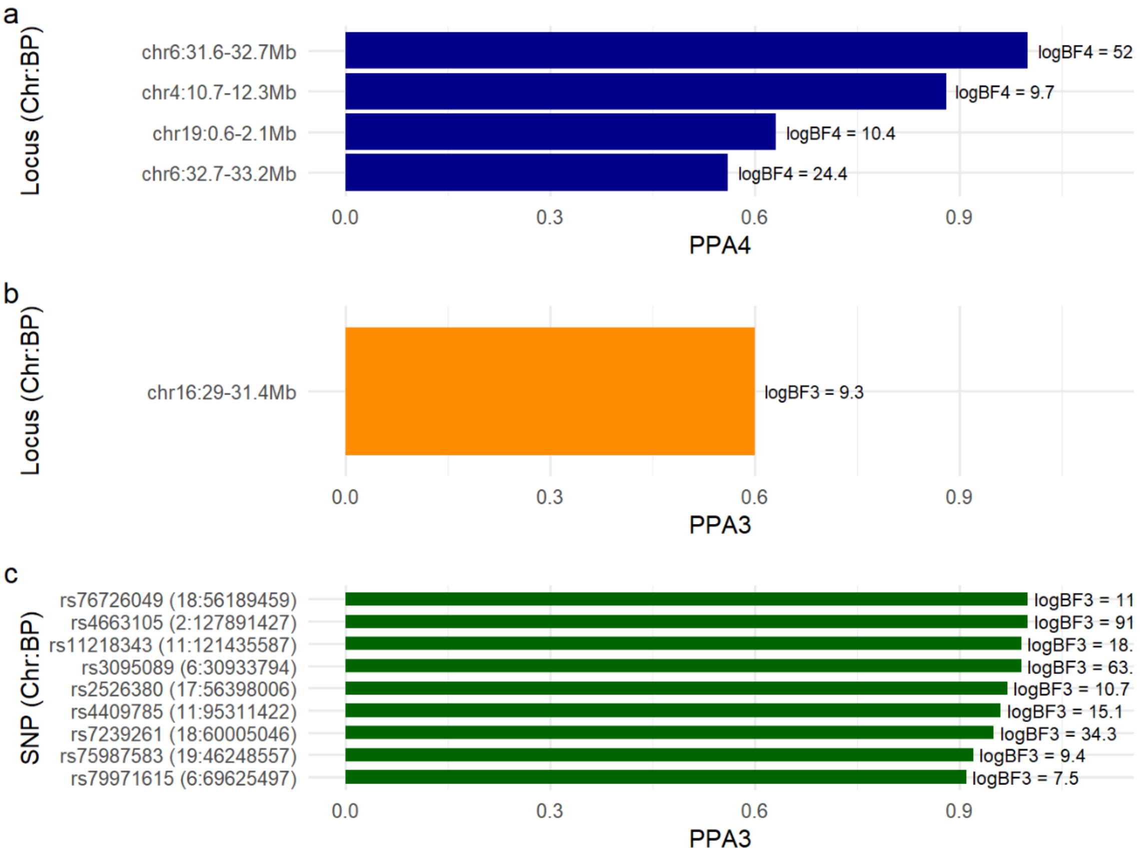

Supplementary Figure 2: Summary of colocalisation analysis between AD and MG

Chr: chromosome, BP: base-pair position, logBF3: The natural logarithm of the regional Bayes factor, which supports the model of a shared association with both phenotypes compared to the null hypothesis. logBF4: The natural logarithm of the regional Bayes factor, which supports the model of two distinct associations (one for each phenotype) compared to the null hypothesis. PPA3: the posterior probability of model 3, PPA4: the posterior probability of model 4. (a) Distinct causal variants (Model 4): This panel shows genomic regions where AD and MG are likely influenced by separate causal variants. Each bar represents a region, and PPA4 is shown on the horizontal axis. Regions with higher PPA4 indicate stronger evidence for distinct associations. The corresponding logBF4 values (annotated on each bar) indicate the strength of support for Model 4 compared to the null. (b) Shared causal variant (Model 3): This panel highlights the genomic region where AD and MG may share a common causal variant. The bar represents the region's posterior probability of Model 3 (PPA3), with the logBF3 (strength of colocalisation) annotated. High PPA3 supports the hypothesis of a single variant influencing both traits. (c) SNPs with high PPA3 (≥ 0.91): This panel presents SNP-level evidence of shared causality. Bars represent SNPs with high posterior probability (PPA3) of being the shared causal variant, ranked by PPA3. While logBF3 varies across loci, the consistently high PPA3 indicates strong confidence in these SNPs as potential shared drivers of AD and MG.
